# Sodium fluoride induces nephrotoxicity via oxidative stress-regulated mitochondrial SIRT3 signaling pathway

**DOI:** 10.1038/s41598-017-00796-3

**Published:** 2017-04-06

**Authors:** Chao Song, Beibei Fu, Jingcheng Zhang, Jiamin Zhao, Mengke Yuan, Wei Peng, Yong Zhang, Haibo Wu

**Affiliations:** 1 College of Veterinary Medicine, Northwest A&F University, Yangling, 712100 Shaanxi China; 2 Key Laboratory of Animal Biotechnology, Ministry of Agriculture, Northwest A&F University, Yangling, 712100 Shaanxi China

## Abstract

Accumulation of mitochondrial reactive oxygen species (mROS) has been implicated in the pathogenesis of fluorosis. As the main mitochondrial deacetylase, SIRT3 is closely associated with oxidative stress. To investigate the role of SIRT3 in response to sodium fluoride (NaF)-induced nephrotoxicity. Our results showed that NaF treatment impaired mitochondrial ultrastructure, decreased cell viability and increased apoptosis in TCMK-1 cells. Oxidative stress, detected by mROS and 8-Hydroxy-2’-deoxyguanosine (8-OHdG) were higher in NaF-treated cells, accompanied by decreased level of reduced glutathione (GSH). NaF reduces manganese superoxide dismutase (SOD2) expression through SIRT3-mediated DNA-binding activity of FoxO3a and decrease SOD2 activity by inhibiting SIRT3-mediated deacetylation. These effects were ameliorated by overexpression of SIRT3. Peroxisome proliferator-activated receptor-coactivator 1a (PGC-1α) interacted with nuclear factor erythroid 2 (NF-E2)-related factor 2 (NRF2) that bound to SIRT3 promoter to regulate SIRT3 expression. The study provides new insights into a critical NRF2/PGC-1α-SIRT3 pathway in response to NaF-induced nephritic oxidative injury. *In vivo* treatment of SIRT3-expressing adenovirus protects against NaF-induced nephritic injury in mice. Moreover, mechanistic study revealed that ERK1/2 activation was associated with increased apoptosis induced by NaF. In conclusion, these data shedding light on new approaches for treatment of NaF-induced nephrotoxicity.

## Introduction

The literature pertaining to the occurrence of environment fluoride (F) and its relationship to human health by researchers is in large quantities and spans a wide variety of disciplines for over 100 years^1^. Although in low dose, it can help strengthen bones and prevent dental caries, however, for instance, due to the wide usage of F in industrial processes, accumulating evidence showed that excessive F exposure causes a variety of adverse effects on soft tissues^2^. The kidney is a multifunctional organ that plays a role in the endocrine, excretory, and hematopoietic systems^3, 4^. It plays an important role in maintaining the homeostasis of the body by selectively excreting or retaining various substances on the basis of specific body needs. The kidney also serves as the main organ for F excretion and as a site of accumulation, hence is vulnerable to F toxicity^5^.

Although several mechanisms have been proposed to explain the sodium fluoride (NaF)-induced toxicity, the mechanisms by which NaF elicits these effects have not been fully elucidated. It is now well accepted that oxidative stress is a common mode of action for NaF both *in vivo* and *in vitro* ^6, 7^. Indeed, fluorosis has been associated with increases in the level of mitochondrial superoxide anion (O_2_^•−^)^8^. Mitochondria are the powerhouse of the cell, and are also the major source and target of reactive oxygen species (ROS)^9^. Mitochondrial manganese superoxide dismutase (SOD2) is the main enzyme responsible for scavenging O_2_^•−^ ^10^. However, the way in which SOD2 is regulated in fluorosis is yet to be elucidated.

SIRT3 (sirtuin 3) is the primary mitochondrial acetyl-lysine deacetylase that modulates various proteins to control mROS level^11^. Residing predominantly in the mitochondria, SIRT3 induces forkhead box O3 (FoxO3a) translocation to the nucleus, thus activates the FoxO3a-dependent antioxidant-encoding gene SOD2^12, 13^. Moreover, SOD2 is a substrate of SIRT3, and the binding of SIRT3 with SOD2 results in the deacetylation and activation of SOD2^14^. However, to date nothing is known about SIRT3 and its presence and role in NaF-induced nephritic oxidative injury.

In this study, our resulted for the first time, that SIRT3 reduction mediates NaF-induced oxidative stress in renal cells. We further demonstrate that SIRT3 mediates SOD2 to regulates mROS level. The reduction of peroxisome proliferator-activated receptor gamma coactivator 1α (PGC-1α), interacting with transcription factor nuclear factor erythroid 2 (NF-E2)-related factor 2 (NRF2), is connected with the decreased SIRT3 in NaF-treated renal cells. The results provide new insights into a key SIRT3 pathway in NaF pathogenesis.

## Results

### NaF-induced cellular injury in TCMK-1 cells

**Figure 1:**
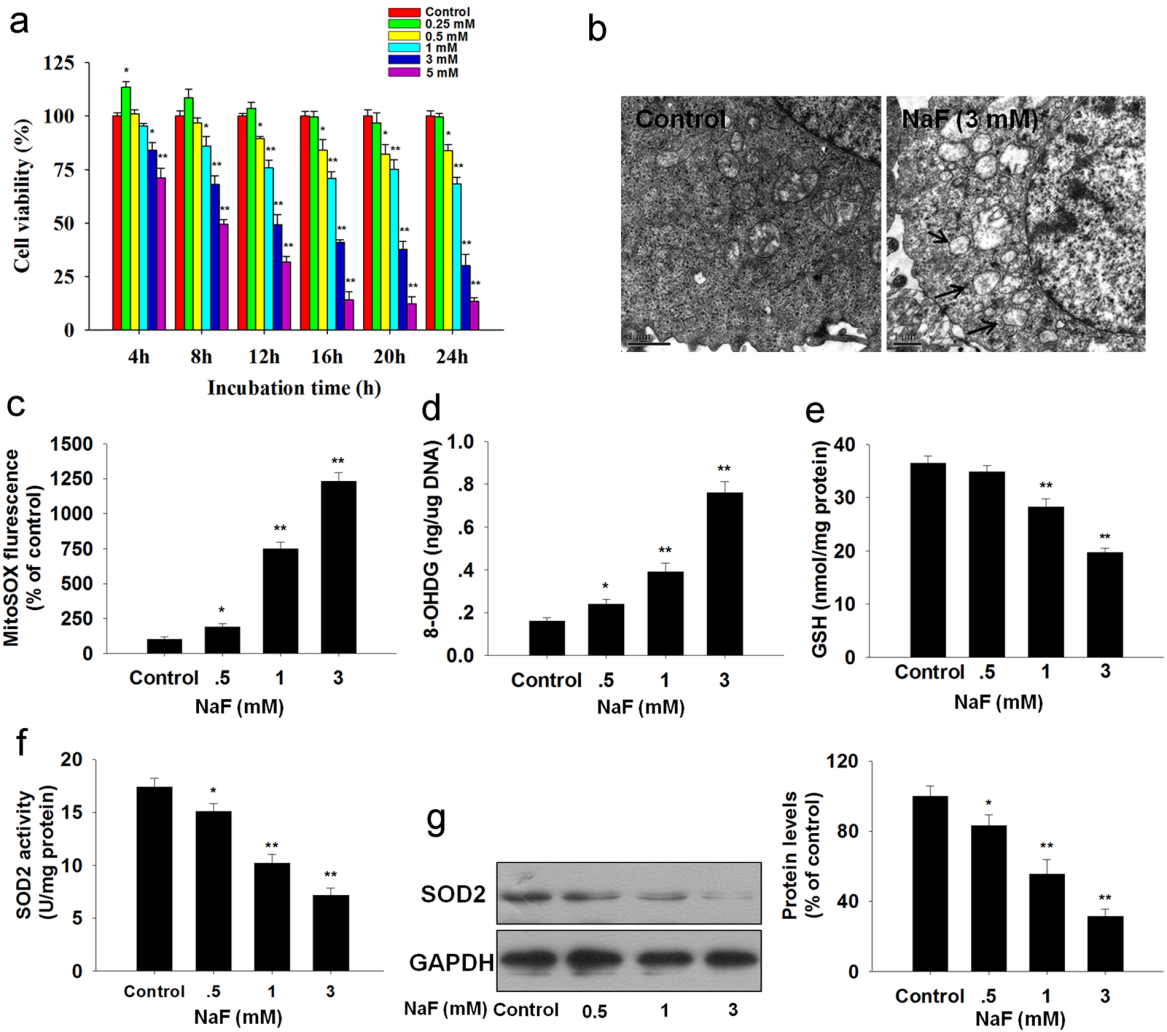
NaF-induced oxidative stress in TCMK-1 cells. (**a**) Cells were incubated with the indicated doses of NaF for the indicated period, and then cell viability was determined using the CCK-8 assay. Cells were incubated with fresh medium in the presence or absence of NaF (3 mM) for 12 h, (**b**) Ultrastructure of TCMK-1 cell. (**c**) The mitochondrial O_2_^•−^ levels. (**d**) 8-OHdG content (a marker of oxidative DNA damage). (**e**) Reduced GSH content. (**f**) SOD2 activity. (**g**) The representative immunoblot and quantification analysis of SOD2. All results are representative of three independent experiments and values are presented as means ± s.d. ^*^p < 0.05, ^**^p < 0.01 versus the control group. Full-length blots/gels are presented in Supplementary Figure S8.

As shown in Fig. 1a, NaF decreased cell viability in a time-dependent and dose-dependent manner in TCMK-1 cells. As shown in Fig. 1b, NaF exposure caused mitochondrial damage. Subsequently, the redox status was evaluated, mitochondrial O_2_^•−^ level was significantly increased in TCMK-1 cells treated with NaF (Fig. 1c). As shown in Fig. 1d, NaF induced DNA damage, indicated by 8-OHdG. Treatment of cells with NaF, also decreased GSH levels significantly (Fig. 1e). Mitochondrial O_2_^•−^ have been implicated in NaF-induced renal cells damage, SOD2 plays a crucial role in the regulation of mitochondrial O_2_^•−^ levels. Our data showed that NaF exposure significantly decreased the activity and expression of SOD2 in a dose-dependent manner (Fig. 1f and g).

**Figure 2:**
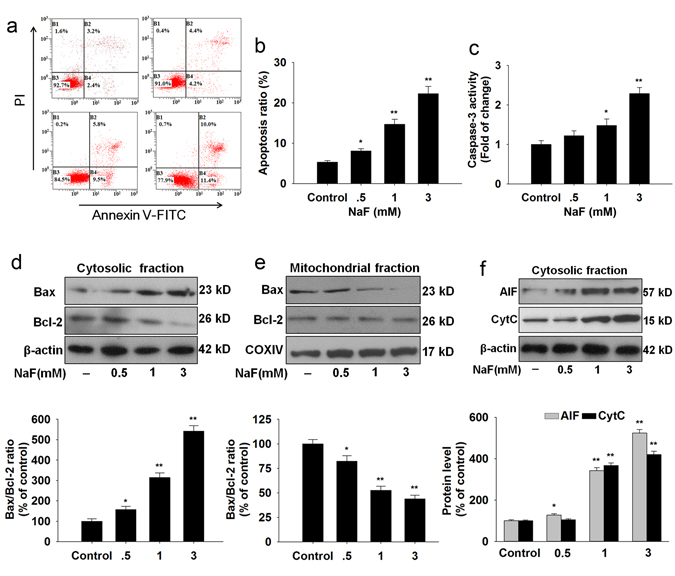
NaF-induced apoptosis in TCMK-1 cells. Cells were incubated with the indicated doses of NaF for 12 h. (**a**) Representative images of flow cytometry by Annexin V-FITC/PI dual staining. (**b**) Apoptotic cells are represented as the percentage of Annexin-V single positive plus Annexin-V/PI double-positive cells. (**c**) Caspase-3 activity was measured using a caspase-3 fluorescent assay kit. Cells were harvested and lysed to detect the cytoplasmic (**d**) and mitochondrial (**e**) levels of Bax and Bcl-2. (**f**) The levels of AIF and CytC in the cytosolic fraction were detected in TCMK-1 cells. All results are representative of three independent experiments and values are presented as means ± s.d. ^*^p < 0.05, ^**^p < 0.01 versus the control group. Full-length blots/gels are presented in Supplementary Figures S9–11.

Apoptosis is closely associated with oxidative stress and plays an important role in NaF pathogenesis. As shown in Fig. 2a and b, treatment with NaF (0.5, 1 and 3 mM) for 12 h showed a significantly higher apoptosis rate over the untreated cells. NaF increased caspase-3 activity (Fig. 2c), an indicator of apoptosis. Moreover, the expressions of apoptosis-related factors including Bax, Bcl-2, AIF and CytC were measured by western blot. The Bax/Bcl-2 ratio after NaF treatment significantly increased in the cytosolic fraction (Fig. 2d), but decreased in the mitochondrial fraction in TCMK-1 cells (Fig. 2e). The cytosolic expression of AIF and CytC were dramatically increased induced by NaF (Fig. 2f). Taken together, these results indicate that NaF-induced oxidative injury in TCMK-1 cells.

### SOD2 overexpression attenuated NaF-induced oxidative injury in TCMK-1 cells

**Figure 3:**
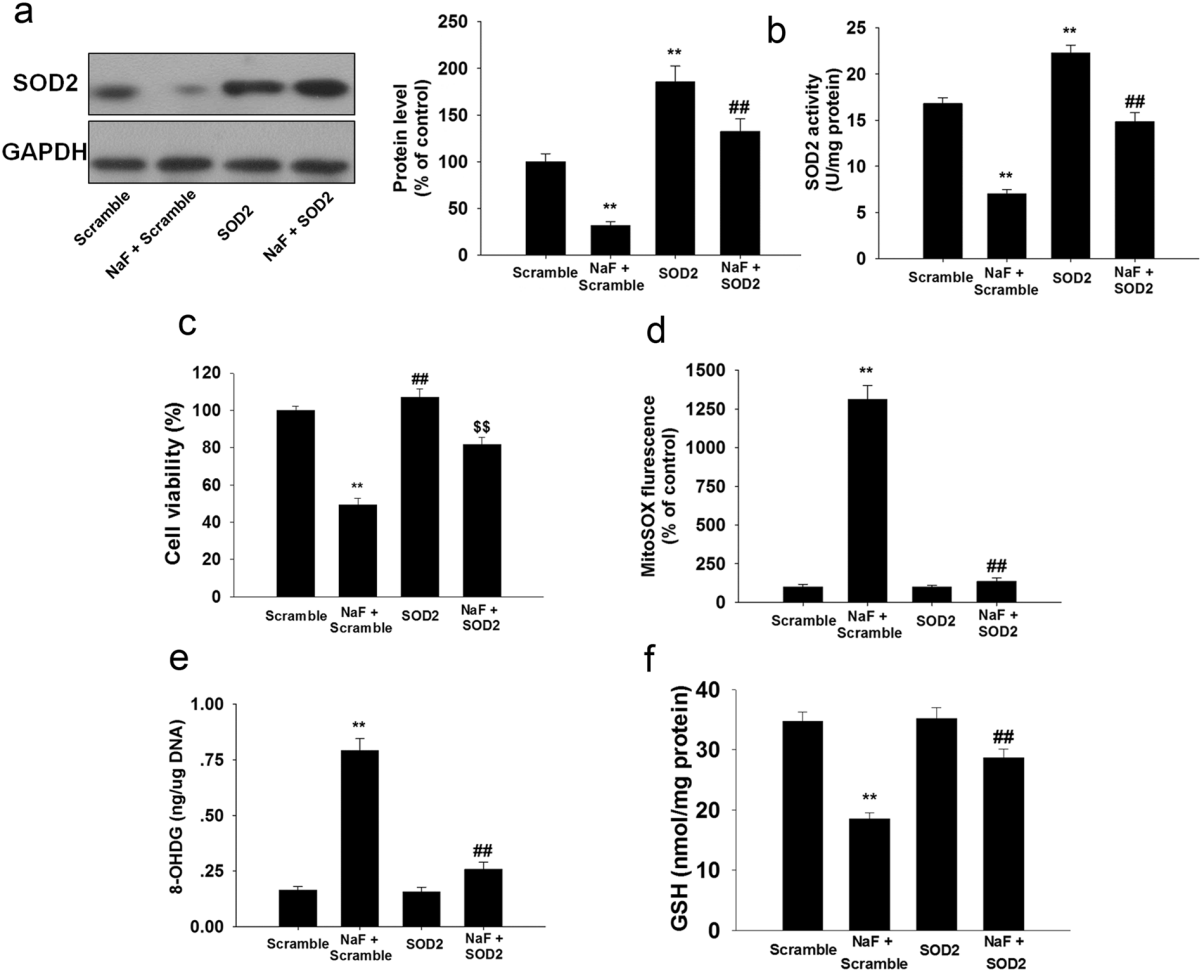
SOD2 overexpression ameliorated NaF-induced oxidative injury in TCMK-1 cells. TCMK-1 cells were transfected with SOD2 expression construct, then, cells were treated with or without NaF of 3 mM for an additional 12 h. (**a**) The representative immunoblot and quantification analysis of SOD2. (**b**) SOD2 activity was measured based on an enzymatic reaction using a SOD2 assay kit. (**c**) Cell viability was measured. (**d**) Mitochondrial-derived O_2_^•−^ production. (**e**) 8-OHdG levels. (**f**) Reduced GSH content. All results are representative of three independent experiments and values are presented as means ± s.d. ^**^p < 0.01 versus scramble group, ^##^p < 0.01 versus the NaF + scramble group. Full-length blots/gels are presented in Supplementary Figure S12.

Next, we examined whether SOD2 overexpression could rescue NaF-induced cellular injury. Overexpression of SOD2 restored the expression (Fig. 3a) and activity of SOD2 (Fig. 3b). Overexpression of SOD2 efficiently increased cell viability (Fig. 3c), suppressed mitochondrial O_2_^•−^ production (Fig. 3d) and 8-OHdG level (Fig. 3e), and reversed GSH content (Fig. 3f) in TCMK-1 cells exposed to NaF.

**Figure 4:**
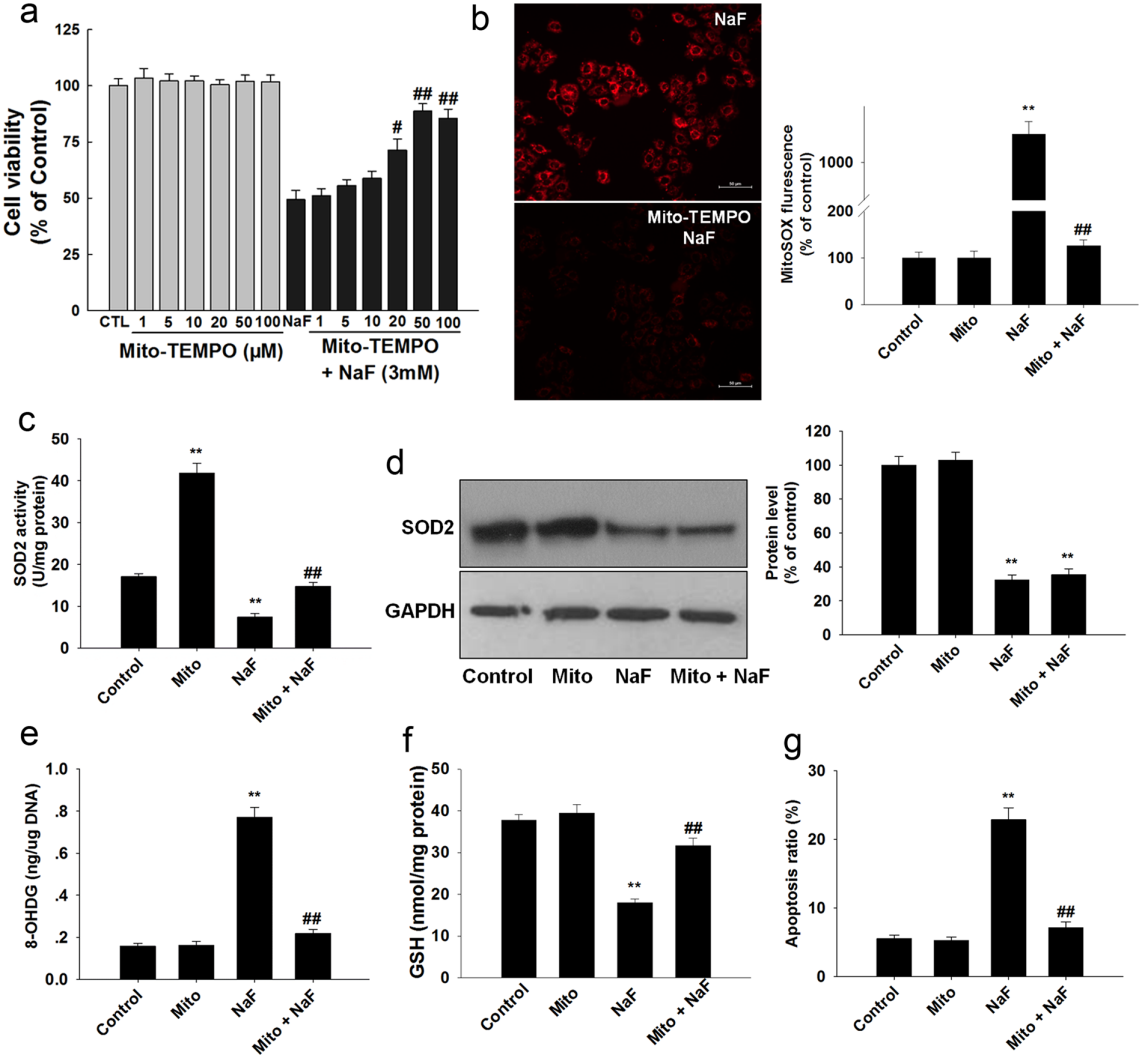
Mito-TEMPO protects against NaF-induced oxidative damage in TCMK-1 cells. TCMK-1 cells were treated with NaF (3 mM) for 12 h in the presence of Mito-TEMPO. (**a**) Cell viability was determined. (**b**) Mitochondrial O_2_^•−^ levels were estimated. (**c**) SOD2 activity was measured. (**d**) Representative blots showing the levels of SOD2. (**e**) The 8-OHDG levels. (**f**) Reduced GSH content. (**g**) Apoptosis ratio. All results are representative of three independent experiments and values are presented as means ± s.d. ^**^p < 0.01 versus control group, ^#^p < 0.05, ^##^p < 0.01 vs. the NaF group. Full-length blots/gels are presented in Supplementary Figure S13.

Moreover, we also incubated TCMK-1 cells with mito-TEMPO (a mitochondrial-targeted SOD mimetic). As shown in Fig. 4a, mito-TEMPO significantly attenuated the adverse effect of NaF on cell viability. 50 μM Mito-TEMPO significantly suppressed mitochondrial-derived O_2_^•−^ generation (Fig. 4b). Interestingly, mito-TEMPO treatment markedly enhanced SOD2 activity (Fig. 4c) but not SOD2 levels (Fig. 4d). The mitochondrial antioxidant mito-TEMPO significantly suppressed NaF-induced oxidative damage (Fig. 4e–g). These data suggest that decreased SOD2 is involved in NaF-induced cellular damage in TCMK-1 cells.

### SIRT3/FoxO3a pathway modulates NaF-induced the decreased SOD2 transcriptionally in TCMK-1 cells

**Figure 5:**
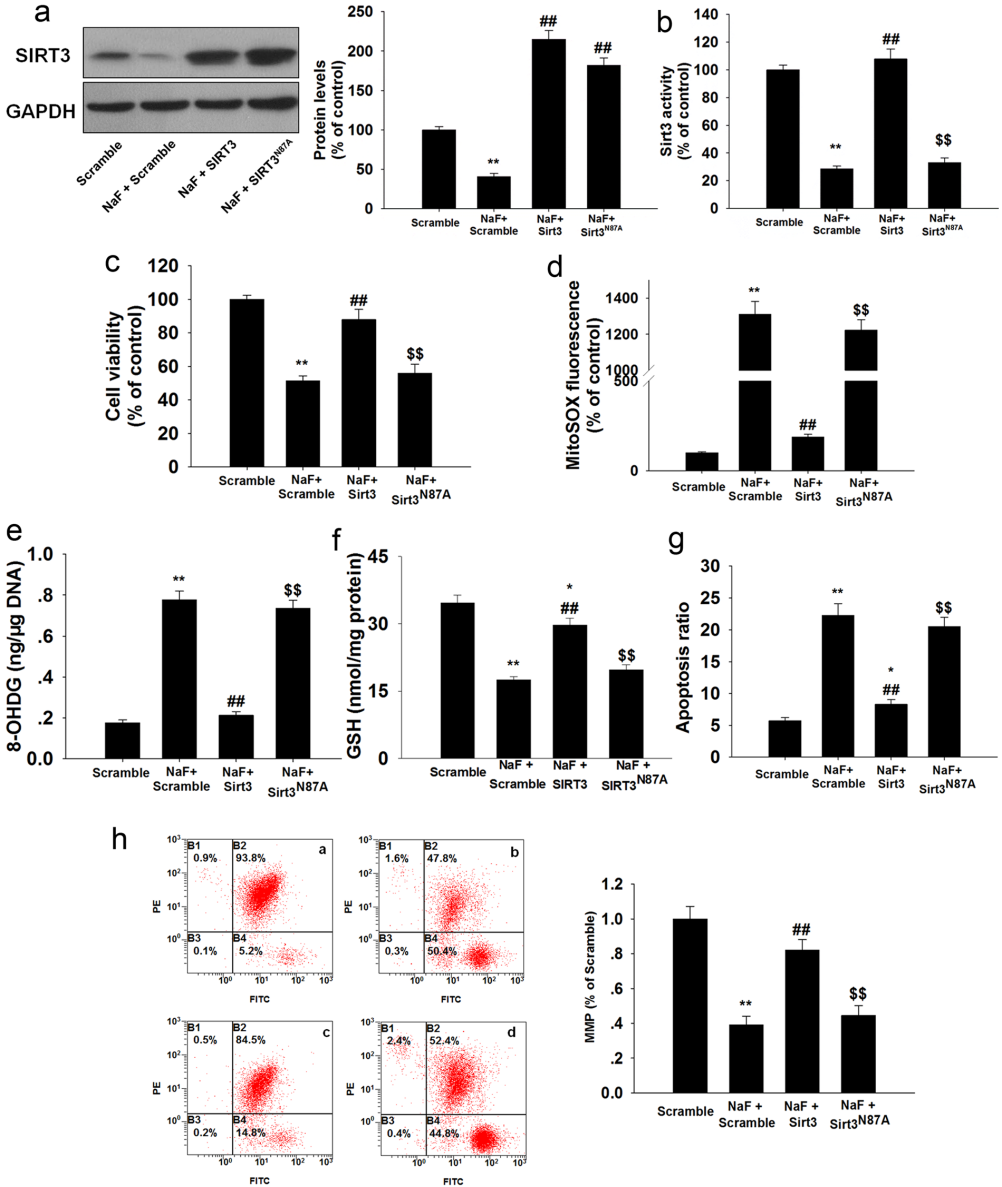
Overexpression of SIRT3 attenuates NaF-induced oxidative injury in TCMK-1 cells. After transfection, cells were treated with or without NaF of 3 mM for 12 h. After the indicated treatments, (**a**) Representative blots showing SIRT3 protein expression in TCMK-1 cells. (**b**) SIRT3 activity was measured. (**c**) Cell viability was measured. The mitochondrial O_2_•^−^ levels (**d**), 8-OHDG level (**e**), Reduced GSH content (**f**), apoptosis (**g**), and MMP (**h**) were determined. All results are presented as means ± s.d. of at least three independent experiments. ^*^p < 0.05, ^**^p < 0.01 versus scramble group, ^##^p < 0.01 versus the NaF + scramble group, ^$$^p < 0.01 versus the NaF + SIRT3 group. Full-length blots/gels are presented in Supplementary Figure S14.

As the main mitochondrial deacetylase, SIRT3 is known to regulate mitochondrial antioxidant function and is closely associated with oxidative stress. Our results showed that NaF exposure not only resulted in a significant decrease in SIRT3 protein levels (Supplementary Fig. S1a) but also decreased SIRT3 activity (Supplementary Fig. S1b). Then we examined if restoration of SIRT3 is sufficient to inhibit NaF-induced oxidative injury and found that SIRT3 overexpression attenuated NaF-induced suppression of SIRT3 protein expression and activity (Fig. 5a and b). Overexpression of SIRT3 significantly increased cell viability (Fig. 5c and Supplementary Fig. S2) attenuated O_2_^•−^ accumulation (Fig. 5d), thus protected cells against NaF-induced oxidative injury (Fig. 5e–g). A decrease in MMP is a hallmark of apoptotic events, SIRT3-overexpressing cells also restored the decreased MMP induced by NaF (Fig. 5h). The deacetylase-deficient SIRT3 mutant (N87A) eliminated the beneficial effects of SIRT3, indicating that SIRT3 deacetylase activity is involved in F-induced oxidative stress. These data indicate that SIRT3 is reduced under NaF treatment and SIRT3 overexpression protects against NaF-induced oxidative injury in TCMK-1 cells.

**Figure 6:**
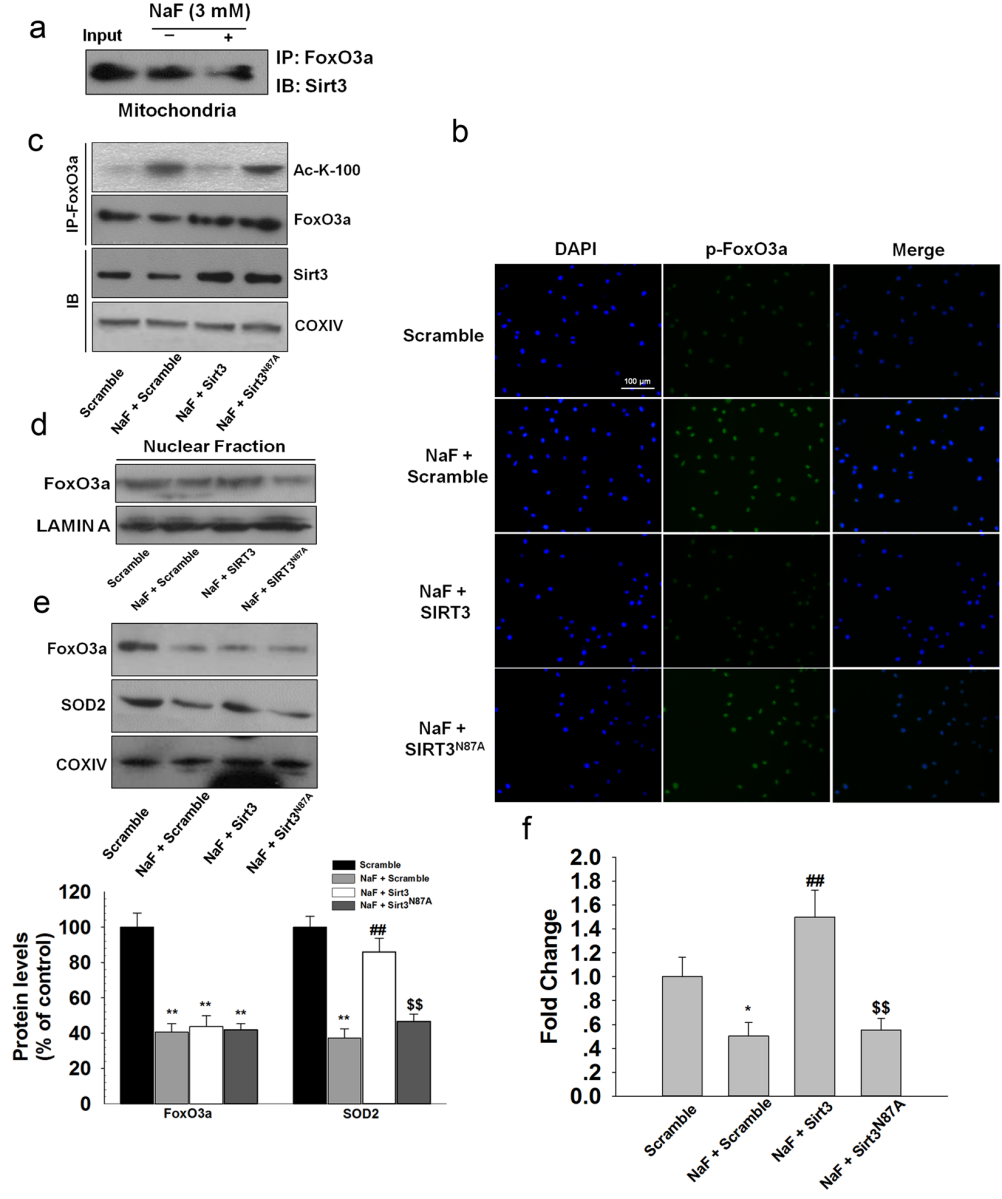
Overexpression of SIRT3 enhanced SOD2 expression through the interaction with FoxO3a in NaF-treated TCMK-1 cells. TCMK-1 cells were transfected with SIRT3 expression constructs (WT or N87A) followed by exposure to NaF (3 mM) for 12 h. (**a**) Mitochondrial fractions were immunoprecipitated with polyclonal antibodies against FoxO3a. Interaction of endogenous SIRT3 and FoxO3a was detected by immunoblotting analysis. (**b**) The expression of phosphorylated FoxO3a was determined by immunocytochemistry using a fluorescence microscope (magnification, × 100). (**c**) FoxO3a acetylation at lysine-100 residue was examined using CO-IP analysis. (**d**) Nuclear location of FoxO3a was measured. (**e**) Expressions of FoxO3a and SOD2 were examined in mitochondrial fraction. (**f**) ChIP analysis was used to examine the binding of FoxO3a to the SOD2 promoter. All results are presented as means ± s.d. of at least three independent experiments. ^*^p < 0.05, ^**^p < 0.01 versus scramble group, ^##^p < 0.01 versus the NaF + scramble group, ^$$^p < 0.01 versus the NaF + SIRT3 group. Full-length blots/gels are presented in Supplementary Figures S15 and 16.

NaF treatment decreased the mRNA level of SOD2, suggesting that the decreased SOD2 was regulated at the transcription level under NaF exposure (Supplementary Fig. S3a). It has been demonstrated that SIRT3-mediated deacetylation of FoxO3a plays an important role in regulating SOD2 expression. NaF treatment decreased FoxO3a expression and increased acetylation of FoxO3 in TCMK-1 cells, the level of acetyl-FoxO3a in TCMK-1 cells correlated inversely with the expression of SIRT3 (Supplementary Fig. S3b and c). Co-IP results indicated that SIRT3 binds directly with FoxO3a in mitochondria, and the binding of SIRT3 to FoxO3a was markedly decreased in NaF-treated cells compared to that of the control (Fig. 6a). Overexpression of SIRT3 decreased the phosphorylation of FoxO3a (Ser 253) and the acetylation levels of FoxO3a in TCMK-1 cells exposed to NaF (Fig. 6b and c; Supplementary Fig. S4a). Since nuclear localization of FoxO3a is essential for its transcriptional activity, SIRT3 overexpression promotes the nuclear translocation of Foxo3a (Fig. 6d; Supplementary Fig. S4b), thus leading to the upregulation of FoxO3a-dependent mitochondrial antioxidant enzyme, SOD2 (Fig. 6e), whereas no significant total protein expression changes were observed in FoxO3a (Fig. 6e).

To further confirm that reduced SOD2 expression is due to decreased bindings of FoxO3a, we performed ChIP assay. We found that the binding of FoxO3a to SOD2 promoter was markedly decreased in NaF-treated cells compared to the control without treatment. SIRT3 overexpression, not SIRT3^N87A^, is able to restore reduced binding of FoxO3a to SOD2 gene promoter induced by NaF treatment (Fig. 6f; Supplementary Fig. S4c). Together, NaF inhibited SIRT3 activity, which consequently decreased the interaction between SIRT3 and FoxO3a, thus decreasing the expression of SOD2, resulting in reduced tolerance towards NaF-induced oxidative stress.

### SIRT3 deacetylases and activates SOD2 in TCMK-1 cells

**Figure 7:**
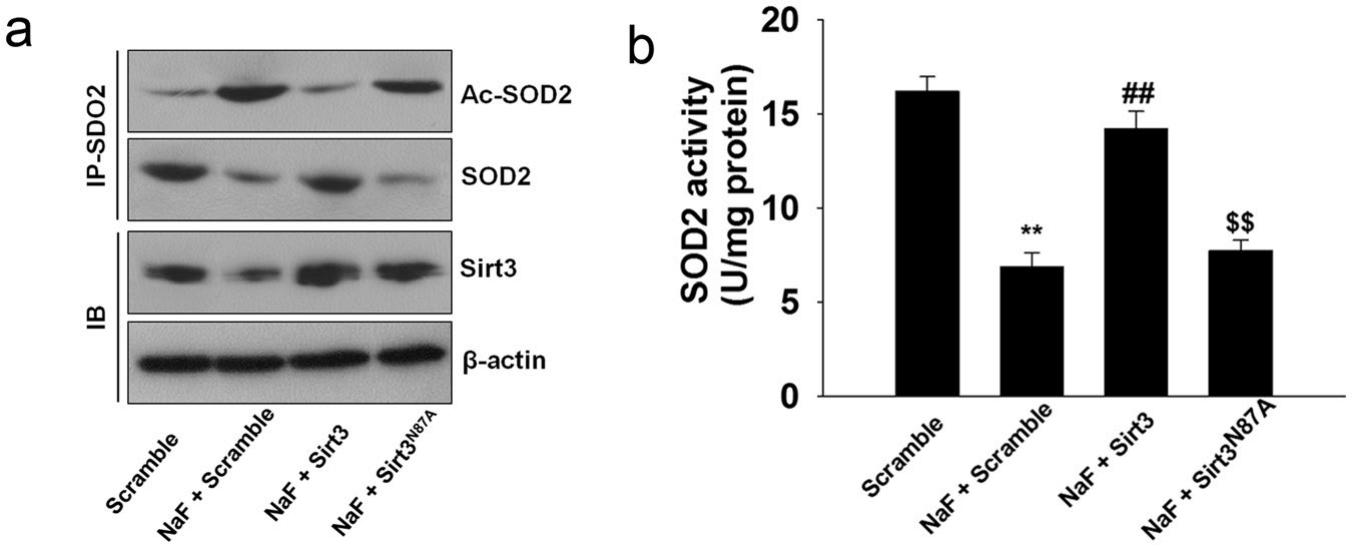
SIRT3 deacetylates and activates SOD2 in TCMK-1 cells. TCMK-1 cells were transfected with SIRT3 expression constructs (WT or N87A) followed by exposure to NaF (3 mM) for 12 h. (**a**) Co-IP confirmed that SIRT3 de-acetylated SOD2 by directly binding to SOD2. (**b**) SOD2 activity was measured. All results are presented as means ± s.d. of at least three independent experiments. ^**^p < 0.01 versus scramble group, ^##^p < 0.01 versus the NaF + scramble group, ^$$^p < 0.01 versus the NaF + SIRT3 group. Full-length blots/gels are presented in Supplementary Figure S17.

SOD2 is also a substrate of SIRT3. The binding of SIRT3 with SOD2 results in the deacetylation and the subsequent activation of its antioxidant activity. Overexpression of SIRT3 blocked the NaF-induced decrease of the acetylated SOD2 and the SOD2 activity. We found that overexpression of SIRT3^N87A^ (a catalytic mutant of SIRT3 lacking deacetylase activity) did not block the increased acetylation of SOD2 and the reduced activity of SOD2 induced by NaF (Fig. 7a and b; Supplementary Fig. 5a and b). These data suggest that the deacetylation of SOD2 is mediated by SIRT3 and the deacetylase activity is required for SOD2 activity.

### PGC-1α interacts with transcription factor NRF2 to regulate SIRT3 expression in TCMK-1 cells

**Figure 8:**
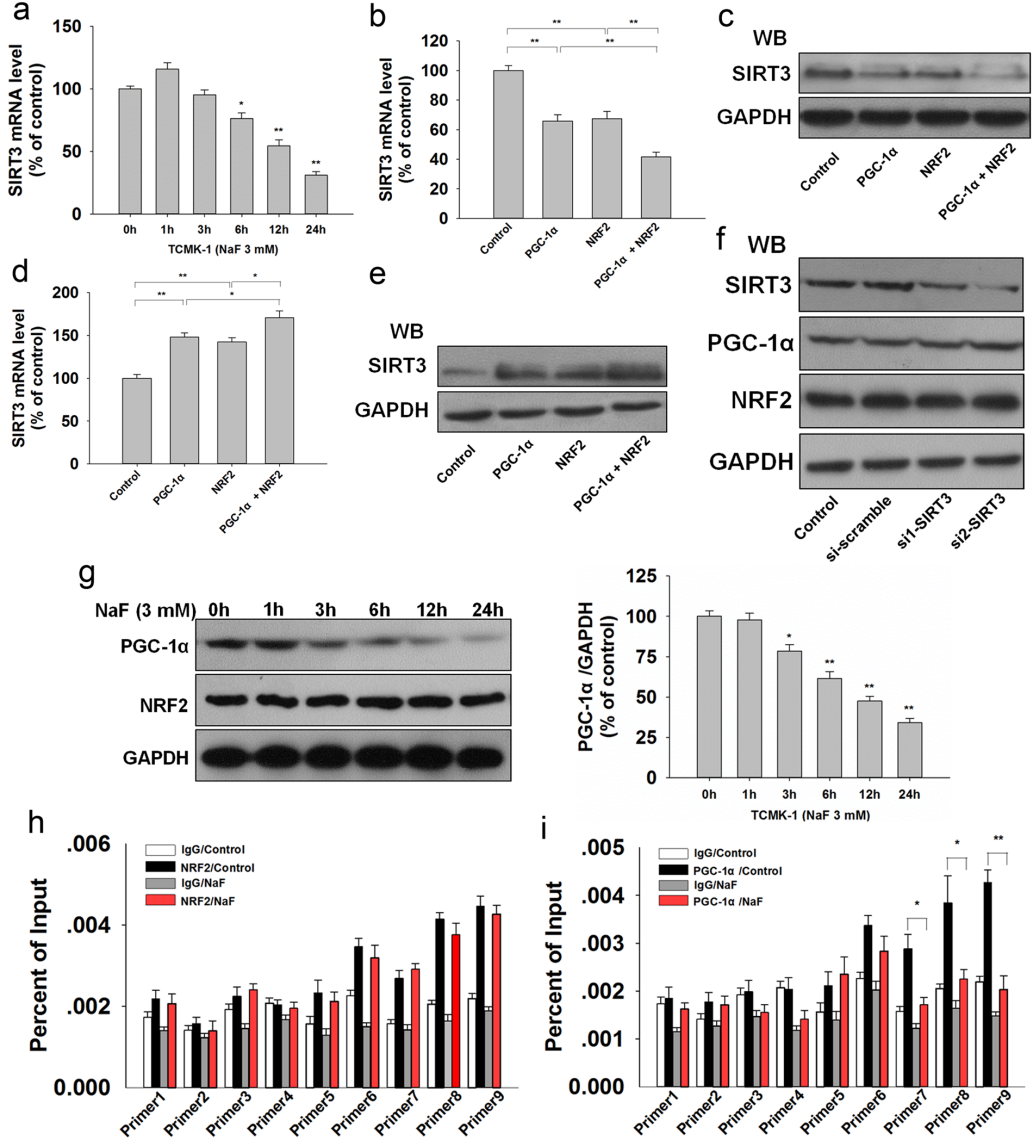
PGC-1α interacts with NRF2 to mediates SIRT3 expression. (**a**) SIRT3 mRNA level was measured by qPCR in TCMK-1 cells treated with 3 mM for different times. Data are presented as means ± s.d. of at least three independent experiments. ^*^p < 0.05, ^**^p < 0.01 versus 0 h. The mRNA and protein expression of SIRT3 were measured with PGC-1α and NRF2 knockdown (**b**,**c**) or overexpression (**d**,**e**), respectively. Data are presented as means ± SD of at least three independent experiments. ^*^p < 0.05, ^**^p < 0.01. (**f**) SIRT3 was knocked down by SIRT3 siRNA transfection, and then the expression of PGC-1α and NRF2 were determined by western blot. (**g**) The expression of PGC-1α and NRF2 were determined in TCMK-1 cells treated with 3 mM for different times. ^*^p < 0.05, ^**^p < 0.01 versus 0 h. ChIP-qPCR was performed with NRF2 (**h**) and PGC-1α (**i**) antibodies in TCMK-1 cells under excessive NaF exposure. Data are presented as means ± s.d. of at least three independent experiments. ^*^p < 0.05, ^**^p < 0.01. Full-length blots/gels are presented in Supplementary Figures S18–20.

Next, we sought to determine which factor mediates the expression of SIRT3. As shown in Fig. 8a, NaF treatment decreased SIRT3 mRNA expression, suggesting that the decreased SIRT3 was regulated at the transcription level under NaF exposure. PGC-1α was reported to interact with transcriptional coactivator to regulate SIRT3 expression. Here, we found that PGC-1α interacted with ERRα and NRF2 in TCMK-1 cells (Supplementary Fig. S6a). To find the transcription factor of SIRT3, we performed a ChIP assay and found that NRF1 or ERRα had a weak binding capacity in the SIRT3 promoter compared with NRF2. Due to the interaction between PGC-1α and NRF2, we also found that PGC-1α had a similar binding pattern with NRF2 in the SIRT3 promoter (Supplementary Fig. S6c–e). As shown in Fig. 8b and c. PGC-1α or NRF2 knockdown decreased the expression of SIRT3, cotransfection of PGC-1α siRNA and NRF2 siRNA could decrease more SIRT3 expression. Moreover, we found that overexpression of PGC-1α or NRF2 increased SIRT3 expression and cotransfection of PGC-1α and NRF2 could induce more expression of SIRT3 (Fig. 8d and e). SIRT3 siRNA treatment efficiently knocked down Sirt3 expression without changing PGC-1α and NRF2 expression levels (Fig. 8f). These results indicated that PGC-1α/NRF2 mediates Sirt3 expression.

Then, we examined which factor was responsible for reduced SIRT3 under NaF exposure. Western blot analysis showed that PGC-1α was decreased under NaF exposure with a similar change pattern of SIRT3 expression, while NRF2 expression was not changed in response to NaF (Fig. 8g). In addition, the ChIP assay indicated that PGC-1α, but not NRF2, decreased its binding with SIRT3 promoter under NaF exposure (Fig. 8h and i; Supplementary Fig. S7a and b). These data revealed that PGC-1α interacts with NRF2 as a transcriptional coactivator to regulate the expression of SIRT3.

### SIRT3 attenuates NaF-induced kidney damage *in vivo*

**Figure 9:**
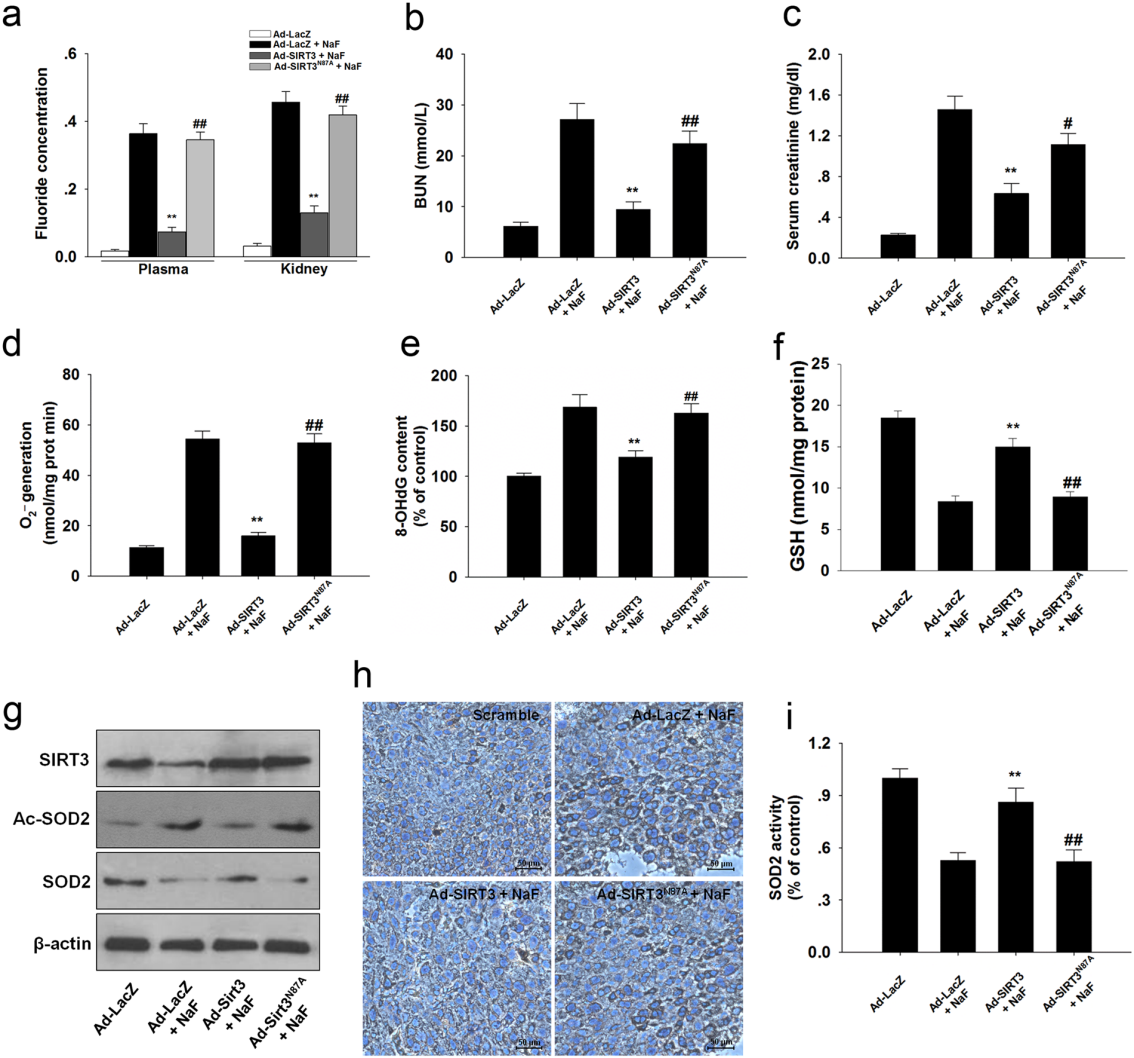
SIRT3 decreased NaF-induced oxidative stress in mice kidney. (**a**) The F concentrations in the serum and renal tissues were measured. Plasma, μg/ml; Kidney, μg /g of dry tissue. (**b**) BUN level. (**c**) Serum creatinine level. (**d**) Mitochondrial O_2_^•−^ level. (**e**) 8-OHdG content. (**f**) Reduced GSH content. (**g**) Representative western blots for SIRT3, Ac-SOD2, and SOD2 in mice kidney. (**h**) Immunohistochemical analysis of SOD2 (acetyl K68) expression in kidney tissue. (**i**) SOD2 activity was determined. Data are mean ± s.d.; n = 6–8. **p < 0.01 versus the Ad-LacZ + NaF group, ^#^p < 0.05, ^##^p < 0.01 versus the Ad-SIRT3 + NaF group. Full-length blots/gels are presented in Supplementary Figure S21.

Next, we investigated the effects of SIRT3 on NaF-induced nephritic injury *in vivo*. Significant accumulation of F was observed in the plasma and kidneys of F-toxicated mice (Fig. 9a). In contrast, pre-infected with SIRT3-expressing adenovirus has brought about significant reduction in the accumulated F-content. The levels of BUN and serum creatinine were markedly elevated after NaF-treatment (Fig. 9b and c). As shown in Fig. 9d–i, SIRT3 overexpression significantly attenuated NaF-induced oxidative damage. However, the infection of mice with SIRT3^N87A^-expressing adenovirus abrogated the protective effect of SIRT3 on NaF-induced nephrotoxicity. These results suggest that SIRT3 effectively prevents NaF-induced oxidative stress in mice kidney.

**Figure 10:**
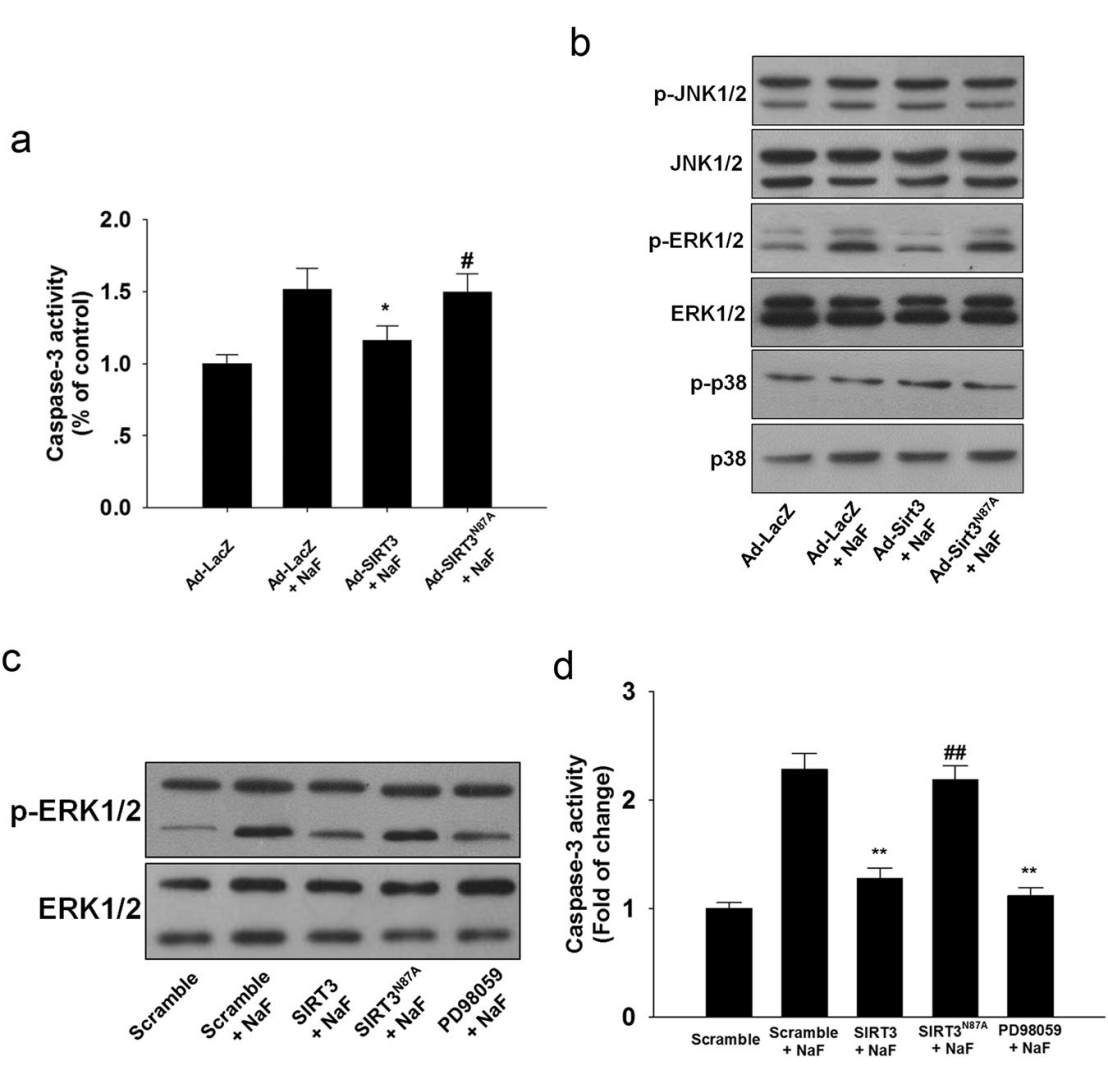
SIRT3 attenuates ERK1/2 activation *in vivo* and *in vitro*. (**a**) Caspase-3 activity in mice kidney. (**b**) Representative western blots for phosphorylated MAPKs (JNK, ERK, p38) in mice kidney. Data are mean ± s.d.; n = 6–8, *p < 0.05 versus the Ad-LacZ + NaF group, ^#^p < 0.05 versus the Ad-SIRT3 + NaF group. (**c**) TCMK-1 cells were pretreated with 20 μM PD98059 for 2 h and then subjected to NaF for 12 h. (**d**) Caspase-3 activity in TCMK-1 cells. Data are mean ± s.d. of at least three independent experiments. **p < 0.01 versus the Scramble + NaF group, ^##^p < 0.01 versus the SIRT3 + NaF group. Full-length blots/gels are presented in Supplementary Figure S22.

Since apoptosis plays an important role in the pathogenetic mechanisms involved fluorosis. Injection of SIRT3 prevented NaF-induced the increase in caspase-3 activity (Fig. 10a) effectively. Activation of mitogen-activated protein kinase (MAPK) has been implicated in NaF-induced apoptosis and they are sensitive to oxidative stress, we therefore investigated the changes of its three family members: JNK1/2, ERK1/2 and p38. Western blotting analysis showed that the expression of phosphorylated ERK1/2 was significantly increased in mice kidney while no change of phosphorylated JNK1/2 and p38 was observed (Fig. 10b). SIRT3 overexpression using SIRT3 expressing adenovirus markedly reduced the phosphorylated ERK1/2 in NaF-treated mice kidney (Fig. 10b). To further investigate the involvement of ERK1/2, we incubated the TCMK-1 cells with NaF. NaF increased the ERK1/2 phosphorylation (Fig. 10c), which was significantly inhibited by PD98059 (a potent ERK1/2 inhibitor). Caspase-3 activity were reduced in NaF-treated TCMK-1 cells in the presence of ERK inhibitor (Fig. 10d).

## Discussion

Elevated consumption of F through intake of water and other dietary components containing high F will impose a burden on the body^15^. Understanding F-induced cytotoxicity will allow us to better understand the pathophysiology of fluorosis. In this study, we found that the major mitochondria deacetylase SIRT3 mediates NaF-induced oxidative damage in TCMK-1 cells. SIRT3 reduction leads to the dysregulation of SOD2, which are involved in the regulation of mROS elimination. Furthermore, our study firstly demonstrates that PGC-1α mediates the SIRT3 expression by interacting with NFR2 and is implicated in mROS accumulation in NaF-induced nephrotoxicity. Notably, treatment with the mitochondria-targeted antioxidant Mito-TEMPO alleviated cellular oxidative stress and increased cell viability. Thus, targeted inhibition of mitochondrial O_2_^•−^ accumulation may be a viable strategy to treat NaF-induced nephrotoxicity.

SIRT3 is the primary mitochondrial deacetylase, and it limits the generation of mitochondrial-derived ROS^16^. The dysregulation of which is the key phenotype of nephritic injury induced by NaF. But whether and how SIRT3 mediates NaF-induced cellular injury to be elucidated. Our results firstly demonstrated that SIRT3 is decreased in a NaF-induced nephrocyte injury. Oxidative injury from excessive O_2_^•−^ accumulation is the major outcome resulting from an overdose of NaF^17^. Mitochondrial antioxidant enzyme SOD2 exerts an important role in eliminating O_2_^•−^ and maintaining redox homeostasis^18^. Current study has shown a link between SIRT3 and mROS level by targeting SOD2 under different pathological and physiological conditions^19^. Mitochondrial O_2_^•−^ are scavenged during calorie restriction because of the SIRT3-mediated deacetylation of SOD2 and the subsequent activation of SOD2^20, 21^. Here, Our study revealed that acetylation in SOD2 is mediated by SIRT3, the loss of which leads to the deacetylation and inactivation of SOD2, which is connected with mROS accumulation in renal cells induced by NaF.

Overexpression of SIRT3 showed antioxidant regulatory effect in liver cells^22^, primary cardiomyocyte^23^, and preadipocytes^24^, indicating that SIRT3 may mediate the antioxidant systems. It has been demonstrated that FoxO3a plays an important role in regulating the SOD2 expression^25^. Nuclear localization of FoxO3a is essential for its transcriptional activity and the transcription of FoxO3a-dependent genes. Increased phosphorylation at Ser253 and acetylation of FoxO3a abolished its nuclear translocation and led to its inactivation, thus reducing the expression of SOD2^26^.

To determine which factor regulates SIRT3 expression, we examined mRNA levels of SIRT3 and found a significant decrease in SIRT3 mRNA under NaF treatment, suggesting that the reduction of SIRT3 was regulated at the transcription level following NaF treatment. Transcriptional coactivator PGC-1α is a crucial regulator in mitochondrial function, it was reported to regulate mitochondrial biogenesis, energy generation and oxidative stress response by interacting with nuclear respiratory factor (NRF1), nuclear erythroid 2 (NF-E2)-related factor 2 (NRF2), and estrogen receptor-related receptor alpha (ERRα)^27–30^. Our results showed that PGC-1α interacted with NRF2 and ERRα in TCMK-1 cells. Furthermore, our data further suggests that PGC-1α interacts with NFR2, not ERRα, as a transcriptional coactivator, which is responsible for the expression and downstream effects of SIRT3 in NaF-induced renal cells injury. Since mitochondrial ROS accumulation and subsequent oxidative stress have been demonstrated to activate a variety of signaling pathways, among which MAPK pathway has been implicated in NaF-induced apoptosis. Activation of JNK1/2 and ERK1/2 contributes to ovarian cells apoptosis under NaF exposure^6, 31^. The present study shows that the levels of phosphorylated ERK1/2 were increased in NaF-treated mouse kidneys; however, phosphorylation of JNK1/2 and p38 remained unaltered. Importantly, our study provides the first evidence that SIRT3 overexpression blocked mitochondrial O_2_^•−^ accumulation, inhibited ERK1/2 activation and prevented apoptosis both *in vivo* and *in vitro*.

**Figure 11:**
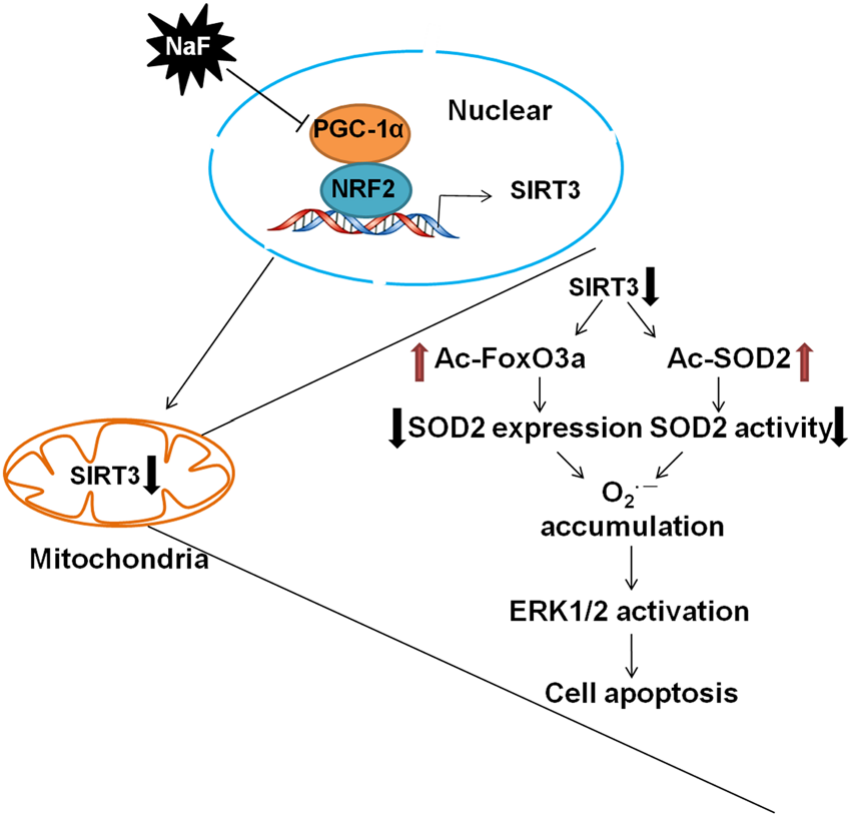
Model of PGC-1α/NRF2-Sirt3 pathway in regulating NaF-induced nephrotoxicity. PGC-1a interacts with NRF2 as a transcriptional coactivator and its reduction decreases Sirt3 transcription. A reduction in Sirt3 decreases the deacetylation of FoxO3a and SOD2, resulting in ROS accumulation. Increased ROS level activates ERK1/2, resulting in cellular apoptosis.

In conclusion, the results of this study provide a novel mechanism by which PGC1α/NFR2-mediated SIRT3 function regulates renal cells damage induced by NaF (Fig. 11). These findings hold important implications for future clinical treatment of fluorosis.

## Materials and Methods

### Reagents and antibodies

All reagents were obtained from Sigma Chemical Co. (St. Louis, MO, USA) unless otherwise indicated. Antibodies against Bax, Bcl-2, β-actin were purchased from Santa Cruz (CA, USA). Antibodies against LAMIN A, COX IV, AIF, CytC, SIRT3, acetylated-lysine, NRF2, GAPDH, phosphorylated and total c-Jun NH2-terminal kinase-1/2 (JNK1/2), extracellular signal-regulated kinase-1/2 (ERK1/2) and p38 kinase were purchased from Cell Signaling Technology (Beverly, MA, USA). Antibodies against SOD2, acetylated-SOD2 (acetyl K68), FoxO3a, phosphorylated-FoxO3a (phospho S253), and PGC-1α were purchased from Abcam (Cambridge, UK).

### Cell culture

Mouse renal tubular epithelial (TCMK-1) cells were purchased from the American Type Culture Collection (ATCC, Manassas, VA, USA). The TCMK-1 cells were cultured in Dulbecco’s Modified Eagle Medium (DMEM, Gibco, USA) that was supplemented with 10% heat-inactivated fetal bovine serum (FBS, Gibco) in a 5% CO_2_ humidified atmosphere at 37 °C.

### CCK-8 assay

Cell viability was determined with Cell Counting Kit-8 Assay Kit (Beyotime, Jiangsu, China). The absorbance was measured with a microplate reader (Epoch, BioTek, Luzern, Switzerland) at a wavelength of 450 nm.

### Transmission electron microscope (TEM) assay

Cells for ultrastructural study were fixed in 2.5% glutaraldehyde and dehydrated in a graded series of ethanol. The sections were obtained and stained with uranyl acetate and lead citrate, and then observed with JEM-1400plus TEM (JEOL, Japan).

### Measurement of O_2_^•−^ generation

The generation of O_2_^•−^ in TCMK-1 cells was determined using MitoSOX red mitochondrial superoxide indicator (Molecular Life Technologies, CA, USA).

### 8-Hydroxy-2’-deoxyguanosine (8-OHdG) measurement

The DNA of each sample was extracted using TIAN amp Genomic DNA Kit (Tian Gen Biotech CO., Ltd., Beijing, China). An oxidative DNA damage enzyme-linked immunosorbent assay (ELISA) Kit (Cell Biolabs, CA, USA) was performed to 8-OHdG following the manufacturer’s instruction. Data were standardized by DNA concentration (ng/mg DNA).

### Determination of reduced glutathione (GSH)

GSH content was measured according to the instruction of a commercially available GSH assay kit (Nanjing Jiancheng Bioengineering Institute, China). GSH content was determined with a spectrophotometer at 420 nm and expressed as nmol GSH/mg protein.

### Caspase-3 activity

Caspase-3 activity was determined using a caspase-3 fluorescent assay kit (Beyotime).

### Annexin V apoptosis detection assay

After treatment, apoptosis detection assay were performed according to the manufacture’s direction strictly (BD Biosciences, CA, USA). Briefly, cells were resuspended in 1× binding buffer in the presence of Annexin V- FITC and propidium iodide, and incubated at room temperature for 30 min in the dark. Analysis was performed by BD LSR II flow cytometry system (Becton Dickinson, NJ, USA).

### Preparation of protein extractions

Cells/Tissues were lysed/homogenized in a lysis buffer containing 0.15 M NaCl, 0.05 M Tris-HCl (pH 7.5), 1% Triton X-100, 0.2% SDS and 3 mM EDTA containing a protease and phosphatase inhibitor cocktail (Sigma-Aldrich). After centrifugation, supernatants were collected for protein analysis. For the cellular protein, mitochondrial and nuclear fractions were prepared using a Mitochondrial or Nuclear extraction kit (Beyotime), respectively.

### Western blot analysis

Western blotting was performed as described previously^32^. In brief, equal amounts of protein were loaded onto 10–15% SDS–polyacrylamide gels and transferred to PVDF membranes. Membranes were incubated with primary antibodies overnight at 4 °C and then developed with horseradish peroxidase-conjugated secondary antibodies for 1 h at room temperature. The corresponding band were revealed by autograph using SuperSignal west pico substrate (Thermo Scientific, IL, USA).

### Plasmid constructs and transfection

The full-length coding sequences of the SOD2, wild-type SIRT3, mutated-SIRT3(N87A), PGC-1α and NRF2 were amplified from TCMK-1 cDNA, and then these sequences were inserted into pEGFP-C1 plasmid by standard molecular cloning methods and confirmed by sequencing. All the primers used for plasmid constructs are listed in the Supporting Information Table S1. The plasmids were transfected with Lipofectamine 2000 (Invitrogen) according to the manufacture’s instruction. The cells were harvested after 24 h transfection and processed for the following experiments.

### SOD2 activity assay

SOD2 activity was assayed with the MnSOD Assay Kit (Beyotime) following the manufacture’s instruction.

### Mitochondrial membrane potential (MMP)

The change in MMP was analyzed by flow cytometry using JC-1 (BioVision, Milpitas, CA, USA). Briefly, after treatment, cells were harvested and resuspended in 500 μL of cell culture media containing 10 μM JC-1 followed by measurement using BD LSR II flow cytometry system.

### Measurement of SIRT3 activity

SIRT3 activity was determined using the CycLex SIRT3 Deacetylase Fluorometric Assay Kit (MBL International Corp. Japan), according to the manufacturer’s instructions. Fluorescence intensity was measured using microplate reader with excitation at 360 nm and emission at 440 nm.

### Quantitative real-time PCR

Cells were extracted using the TRIzol (Invitrogen). One microgram of total RNA was reverse transcribed to cDNA using PrimeScript RT reagent Kit (Takara, Otsu, Shiga, Japan). Quantitative real-time PCR (qPCR) using an ABI StepOnePlus PCR system (Applied Biosystems), SYBR Premix ExTaq II (TaKaRa). The primer sequences for qPCR were listed in Supplementary Table S2.

### Immunoprecipitation

Immunoprecipitation (IP) was conducted according to methods described previously with a few modifications^33^. Lysates were clarified by centrifugation at 14,000 × g for 15 min and adjusted to the same protein concentration with the respective lysis buffer for IP. Briefly, protein extracts were incubated with antibody overnight at 4 °C and then fresh protein A/G-conjugated beads (Santa Cruz) were added for storage overnight at 4 °C. Finally, the beads were washed three times with the same lysis buffer and eluted using the sample loading buffer and analyzed by immunoblotting.

### Chromatin Immunoprecipitation (ChIP) assay

A ChIP assay was performed using the Pierce Agarose ChIP kit as described previously^34^. Briefly, TCMK-1 cells were cross-linked with 1% (wt/vol) formaldehyde for 15 min at room temperature followed by 125 mM glycine treatment to stop the cross-linking. Genomic DNA was isolated and sheared to average lengths of 300–500 bp by ultrasonic waves and 1% of the supernatant was regarded as input. Precipitated genomic DNA was amplified by real-time PCR with primers listed in Supplementary Tables S3 and S4.

### RNA interference

Cells were transfected with targeted siRNAs with Lipofectamine RNAiMAX (Invitrogen) at a final concentration of 10 μM for 48 h.

### Animal ethics

All procedures were performed in accordance with the Animal Management Rules of the Ministry of Health of the People’s Republic of China, and were approved by the by the Animal Care Commission of the College of Veterinary Medicine, Northwest A&F University.

Seven-week-old female Kunming mice were purchased from the experimental animal center of the fourth military medical university. Mice were housed under specific pathogen-free conditions at 25 °C, and maintained under a 12 h light/dark cycle with ad libitum access to food (the total content of F is 0.67 ± 0.08 mg/kg) and water.

### Animal treatments

The AdEasy^TM^ adenovirial vector system was utilized to construct the adenovirus expression vectors. Each mice was administrated with 1 × 10^9^ pfu particles of recombinant adenoviral construct expressing LacZ, SIRT3, and SIRT3 (N87A) via caudal vein injection. At 2 days later, all mice except control were injected intraperitoneally with NaF (8 mg/kg) for 14 d. The control mice received an equal volume of normal saline.

### Estimation of Fluoride

The fluoride levels in the plasma and kidney were estimated measured according to a potentiometric method^1^ using Thermo Orion 720A+ with fluoride ion sensitive electrode 9609BNWP (Thermo, USA).

### Renal function

Blood urea nitrogen (BUN) and serum creatinine were measured with colorimetric detection kits (Nanjing Jiancheng Bioengineering Institute, Nanjing, China), according to the manufacturer’s instructions respectively.

### *In vivo* superoxide detection

The assays of O_2_^•−^ production in kidneys were determined by means of spectrophotometry with a commercially available kit (Nanjing Jiancheng Bioengineering Institute) as previously described^35^.

### Immunohistochemical analyses

Fixed mice kidneys were embedded in paraffin and were sectioned at a thickness of 5 μm, and immunohistochemical staining was performed with acetylated-SOD2 (1:100) antibody.

### Statistical analysis

Raw data were analyzed using SPSS 19.0 software (Chicago, IL, USA). All of the experiments were performed a minimum of three times and data are expressed as the mean ± standard deviation (s.d.). ANOVA and Bonferroni correction were used to determine differences between different groups. Student’s t-test was used to determine differences between two groups. p < 0.05 was considered statistically significant.

## Electronic supplementary material

Supplementary information

## References

[1] Mahaboob Basha P, Saumya SM (2013). Suppression of mitochondrial oxidative phosphorylation and TCA enzymes in discrete brain regions of mice exposed to high fluoride: amelioration by Panax ginseng (Ginseng) and Lagerstroemia speciosa (Banaba) extracts. Cell Mol Neurobiol.

[2] Ameeramja J (2016). Tamarind seed coat ameliorates fluoride induced cytotoxicity, oxidative stress, mitochondrial dysfunction and apoptosis in A549 cells. J Hazard Mater.

[3] Hingorani S (2016). Renal Complications of Hematopoietic-Cell Transplantation. N Engl J Med.

[4] Kurt B, Kurtz A (2015). Plasticity of renal endocrine function. Am J Physiol Regul Integr Comp Physiol.

[5] Chattopadhyay A, Podder S, Agarwal S, Bhattacharya S (2011). Fluoride-induced histopathology and synthesis of stress protein in liver and kidney of mice. Arch Toxicol.

[6] Geng Y (2014). Sodium fluoride activates ERK and JNK via induction of oxidative stress to promote apoptosis and impairs ovarian function in rats. J Hazard Mater.

[7] Varol E, Varol S (2012). Effect of fluoride toxicity on cardiovascular systems: role of oxidative stress. Arch Toxicol.

[8] Garcia-Montalvo EA, Reyes-Perez H, Del Razo LM (2009). Fluoride exposure impairs glucose tolerance via decreased insulin expression and oxidative stress. Toxicology.

[9] Shabalina IG (2014). ROS production in brown adipose tissue mitochondria: the question of UCP1-dependence. Biochim Biophys Acta.

[10] Storz P (2011). Forkhead homeobox type O transcription factors in the responses to oxidative stress. Antioxid Redox Signal.

[11] Pi H (2015). SIRT3-SOD2-mROS-dependent autophagy in cadmium-induced hepatotoxicity and salvage by melatonin. Autophagy.

[12] Padmaja Divya S (2015). Arsenic Induces Insulin Resistance in Mouse Adipocytes and Myotubes Via Oxidative Stress-Regulated Mitochondrial Sirt3-FOXO3a Signaling Pathway. Toxicol Sci.

[13] Sundaresan NR (2009). Sirt3 blocks the cardiac hypertrophic response by augmenting Foxo3a-dependent antioxidant defense mechanisms in mice. J Clin Invest.

[14] Wei L (2015). Oroxylin A inhibits glycolysis-dependent proliferation of human breast cancer via promoting SIRT3-mediated SOD2 transcription and HIF1alpha destabilization. Cell Death Dis.

[15] Sun Z (2010). Effects of sodium fluoride on hyperactivation and Ca2+ signaling pathway in sperm from mice: an *in vivo* study. Arch Toxicol.

[16] Bell EL, Emerling BM, Ricoult SJ, Guarente L (2011). SirT3 suppresses hypoxia inducible factor 1alpha and tumor growth by inhibiting mitochondrial ROS production. Oncogene.

[17] Izquierdo-Vega JA, Sanchez-Gutierrez M, Del Razo LM (2008). Decreased *in vitro* fertility in male rats exposed to fluoride-induced oxidative stress damage and mitochondrial transmembrane potential loss. Toxicol Appl Pharmacol.

[18] Zhang X (2016). PGC-1alpha/ERRalpha-Sirt3 Pathway Regulates DAergic Neuronal Death by Directly Deacetylating SOD2 and ATP Synthase beta. Antioxid Redox Signal.

[19] Chen Y (2011). Tumour suppressor SIRT3 deacetylates and activates manganese superoxide dismutase to scavenge ROS. EMBO Rep.

[20] Hebert AS (2013). Calorie restriction and SIRT3 trigger global reprogramming of the mitochondrial protein acetylome. Mol Cell.

[21] Qiu X, Brown K, Hirschey MD, Verdin E, Chen D (2010). Calorie restriction reduces oxidative stress by SIRT3-mediated SOD2 activation. Cell Metab.

[22] Kendrick AA (2011). Fatty liver is associated with reduced SIRT3 activity and mitochondrial protein hyperacetylation. Biochem J.

[23] You, J. *et al*. Receptor-Interacting Protein 140 represses Sirtuin 3 to facilitate hypertrophy, mitochondrial dysfunction and energy metabolic dysfunction in cardiomyocytes. *Acta Physiol* (*Oxf*) (2016). 10.1111/apha.12800 27614093

[24] Kim HL (2015). Platycodon grandiflorum A. De Candolle Ethanolic Extract Inhibits Adipogenic Regulators in 3T3-L1 Cells and Induces Mitochondrial Biogenesis in Primary Brown Preadipocytes. J Agric Food Chem.

[25] Tao R (2010). Sirt3-mediated deacetylation of evolutionarily conserved lysine 122 regulates MnSOD activity in response to stress. Mol Cell.

[26] Sunters A (2006). Paclitaxel-induced nuclear translocation of FOXO3a in breast cancer cells is mediated by c-Jun NH2-terminal kinase and Akt. Cancer Res.

[27] Cherry AD, Suliman HB, Bartz RR, Piantadosi CA (2014). Peroxisome proliferator-activated receptor gamma co-activator 1-alpha as a critical co-activator of the murine hepatic oxidative stress response and mitochondrial biogenesis in Staphylococcus aureus sepsis. J Biol Chem.

[28] St-Pierre J (2006). Suppression of reactive oxygen species and neurodegeneration by the PGC-1 transcriptional coactivators. Cell.

[29] Finck BN, Kelly DP (2006). PGC-1 coactivators: inducible regulators of energy metabolism in health and disease. J Clin Invest.

[30] Gleyzer N, Vercauteren K, Scarpulla RC (2005). Control of mitochondrial transcription specificity factors (TFB1M and TFB2M) by nuclear respiratory factors (NRF-1 and NRF-2) and PGC-1 family coactivators. Mol Cell Biol.

[31] Nguyen Ngoc TD (2012). Sodium fluoride induces apoptosis in mouse embryonic stem cells through ROS-dependent and caspase- and JNK-mediated pathways. Toxicol Appl Pharmacol.

[32] Wu H (2014). Vitamin C enhances Nanog expression via activation of the JAK/STAT signaling pathway. Stem Cells.

[33] Tseng AH, Shieh SS, Wang DL (2013). SIRT3 deacetylates FOXO3 to protect mitochondria against oxidative damage. Free Radic Biol Med.

[34] Zhou X (2014). Resveratrol regulates mitochondrial reactive oxygen species homeostasis through Sirt3 signaling pathway in human vascular endothelial cells. Cell Death Dis.

[35] Gao G (2012). Ovarian dysfunction and gene-expressed characteristics of female mice caused by long-term exposure to titanium dioxide nanoparticles. J Hazard Mater.

