# Author Correction: Sodium fluoride induces nephrotoxicity via oxidative stress-regulated mitochondrial SIRT3 signaling pathway

**DOI:** 10.1038/s41598-018-25422-8

**Published:** 2018-05-11

**Authors:** Chao Song, Beibei Fu, Jingcheng Zhang, Jiamin Zhao, Mengke Yuan, Wei Peng, Yong Zhang, Haibo Wu

**Affiliations:** 1 College of Veterinary Medicine, Northwest A&F University, Yangling, 712100 Shaanxi China; 2 Key Laboratory of Animal Biotechnology, Ministry of Agriculture, Northwest A&F University, Yangling, 712100 Shaanxi China

Correction to: *Scientific Reports* 10.1038/s41598-017-00796-3, published online 06 April 2017

This Article contains an error in the Discussion section:

“Here, Our study revealed that acetylation in SOD2 is mediated by SIRT3, the loss of which leads to the deacetylation and inactivation of SOD2, which is connected with mROS accumulation in renal cells induced by NaF.”

should read:

“Here, our study revealed that acetylation in SOD2 is mediated by SIRT3, the loss of which leads to the acetylation and inactivation of SOD2, which is connected with mROS accumulation in renal cells induced by NaF.”

This message is conveyed correctly elsewhere in the Article, and as such the conclusions are unaffected. The authors apologize for the error.

