# Supplementary material for: Sodium fluoride induces nephrotoxicity via oxidative stress-regulated mitochondrial SIRT3 signaling pathway
Source: Sci Rep. 2017 Apr 6;7:672. doi: 10.1038/s41598-017-00796-3 (PMC5429606; doi:10.1038/s41598-017-00796-3)
Supplement: Supplementary file 1 — Supplementary information [file 41598_2017_796_MOESM1_ESM.pdf]

Supporting Information

Title: Sodium fluoride induces nephrotoxicity via oxidative stress-regulated mitochondrial SIRT3 signaling pathway

Authors: Chao Song[a, b, 1], Beibei Fu[a, b,1], Jingcheng Zhang[a, b], Jiamin Zhao[a, b], Mengke Yuan[a, b], Wei Peng[a, b], Yong Zhang [a, b,*], Haibo Wu [a, b,*]

Author affiliations: [a] College of Veterinary Medicine, Northwest A&F University, Yangling 712100, Shaanxi, China.

[b] Key Laboratory of Animal Biotechnology, Ministry of Agriculture, Northwest A&F University, Yangling 712100, Shaanxi, China.

[1]These authors contributed equally to this study.

Corresponding author: *Correspondence should be addressed to Haibo Wu or Yong Zhang.

Tel.: +86 29 87080092

Fax: +86 29 87080092

E-mail: hbwu029@nwsuaf.edu.cn (H.W) or zhangy1956@sina.com (Y.Z)

This work was funded by the State Key Program (No. 31530075) of National Natural Science Foundation of China and China Postdoctoral Science Foundation Grant (No. 2016M590978)

Supplementary Tables

Supplementary Table S1 primers used for plasmid constructs

Primers	Primer Sequence (5'→3')
SOD2	F 5'- AATCTCGAGGCAGCGGTCGTGTAAACCT -3'<br>R 5'- TCAGGTACCCTCAATGTGGCCGTGAGTG -3'
SIRT3	F 5'- CTTCTCGAGGTAGGGTGGTGGTCAT -3'<br>F 5'- CCAGGTACCTCTTGCTGGACATAGGA -3'
SIRT3(N87A)	F 5'- TACACAGGCCATCGACGGGCTT -3'<br>F 5'- TCTCTCCGGCCCGTCGATGTT -3'
PGC-1α	F 5'- GAACTCGAGCTGGTTGCCTGCATGAGT -3'<br>R 5'- GCTGGATCCTCTTGGGAAAGGACACG -3'
NRF2	F 5'- TACCTCGAGAGCCCTACCACAGCGTC -3'<br>F 5'- ACAGGTACCCTCCATCCTCCCGAACC -3'

Supplementary Table S2 Primer sequences used for qPCR

Primers	Primer Sequence (5'→3')
SOD2	F 5'-CAGACCTGCCTTACGACTATGG-3'<br>R 5'-CTCGGTGGCGTTGAGATTGTT -3'
SIRT3	F 5'-ATCCCGGACTTCAGATCCCC-3'<br>R 5'-CAACATGAAAAAGGGCTTGGG-3'
GAPDH	F 5'-AGGTCGGTGTGAACGGATTTG-3'<br>F 5'-TGTAGACCATGTAGTTGAGGTCA-3'

Supplementary Table S3 ChIP-qPCR primers of SOD2

Primers	Primer Sequence (5'→3')
SOD2	F 5'-TTATGGAAACATTTGATAGCCACTGCTTCTTAGAC-3'
	R 5'-CGCGTGCTTGCTACAGCCACGC-3'

Supplementary Table S4 ChIP-qPCR primers of SIRT3

Primers	Forward primer	Reverse primer
Primer1	ACAGCGTCAACTCCCACT	ATCCGTTTCTTCACATTAGG
Primer2	GTATGCTATTTCGGTGTCC	GAAGGCATTGGTTGTGAC
Primer3	CGAAATAGCATACTTCATACC	GACAATGTCCAGCAGTTAA
Primer4	GAACAATTACGAATTGCTG	GAGTTTTAACTGCTGGACA
Primer5	AGCTGGTAAGGCTGGAAC	AGGGTCAGGGATGTGGAG
Primer6	TAGCGGAAGTCAAGAACG	AGTCAGAACCGGCAGGGAT
Primer7	GTTGCGGTCGTCAAGGTA	GGTTTCAGAGGACCCAAGT
Primer8	TTGAGTAGGCGGGTGATA	TGAGGCATTAAAGAGTAGAGG
Primer9	GCCTACTCAAGGAGGTCG	TGTTTATGCCTGGTGCTG

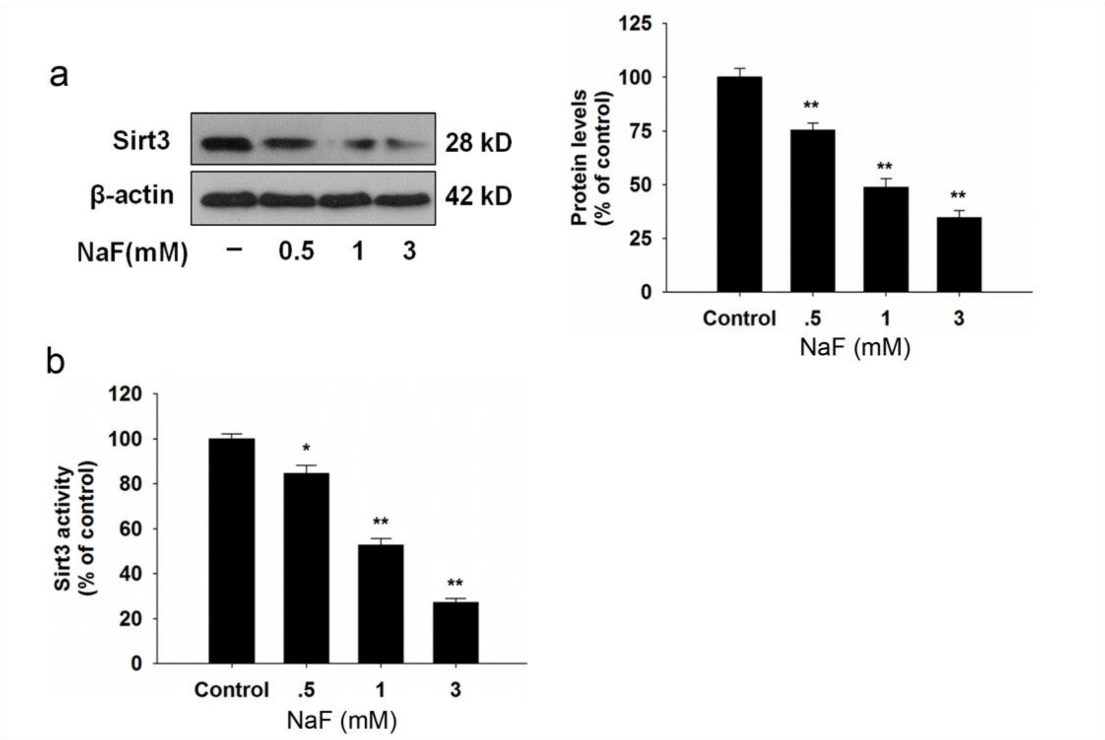

Supplementary Figure S1 Effects of NaF on SIRT3 expression and activity in TCMK-1 cells. Cells were incubated with the indicated doses of NaF for 12h. (a) A representative immunoblot and quantification analysis of SIRT3. (b) SIRT3 activity was measured based on an enzymatic reaction using a SIRT3 assay kit. Data are presented as means ± s.d. of at least three independent experiments. $^{*}p < 0.05$, $^{**}p < 0.01$ versus control.

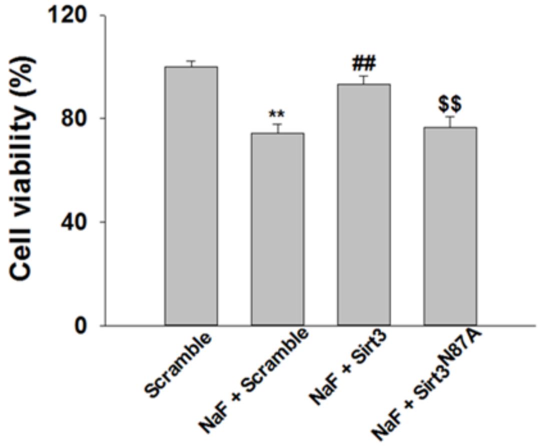

Supplementary Figure S2 Overexpression of SIRT3 attenuates NaF-induced oxidative injury in TCMK-1 cells. After transfection, TCMK-1 cells were incubated with 1 mM of NaF for 12h. Cell viability was measured. $^{**}p < 0.01$ versus scramble group, $^{\#\#}p < 0.01$ versus the NaF + scramble group, $^{\$\$}p < 0.01$ versus the NaF + SIRT3 group.

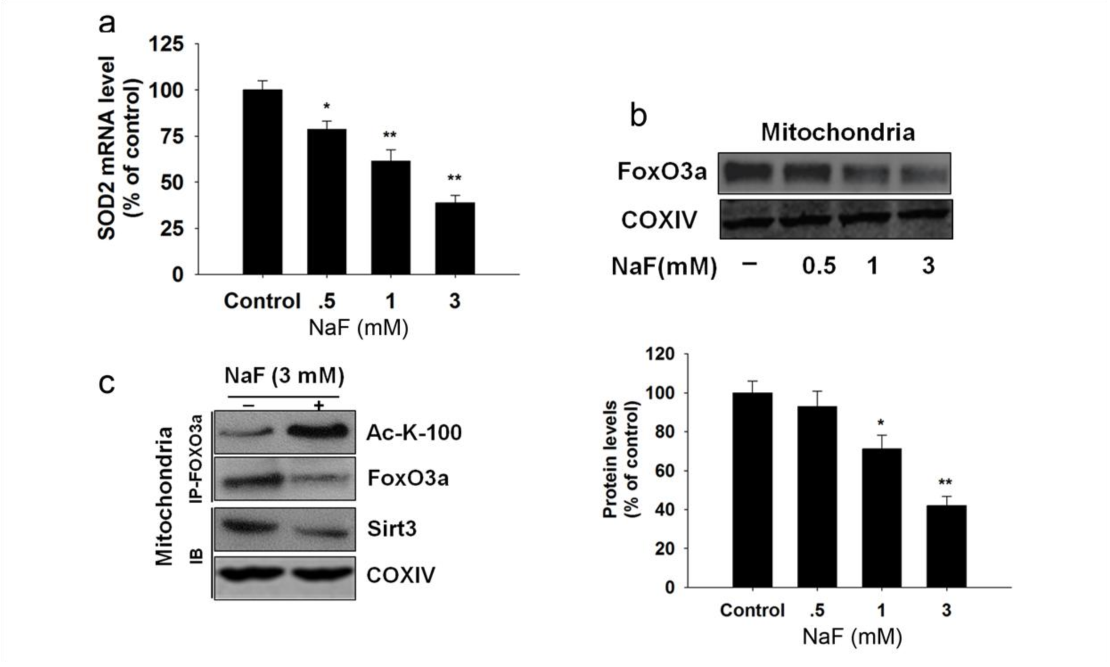

Supplementary Figure S3 NaF treatment inhibited SIRT3 target proteins. Cells were incubated with the indicated doses of NaF for 12h. (a) SOD2 mRNA level was measured by qPCR in TCMK-1 cells. (b) FoxO3a expression was examined using

immunoblotting. (c) Immunoprecipitation (IP) was conducted to examine FoxO3a acetylation at lysine-100 residue. Data are presented as the mean ± s.d. of three independent experiments. $^{*}p < 0.05$, $^{**}p < 0.01$ versus the control group.

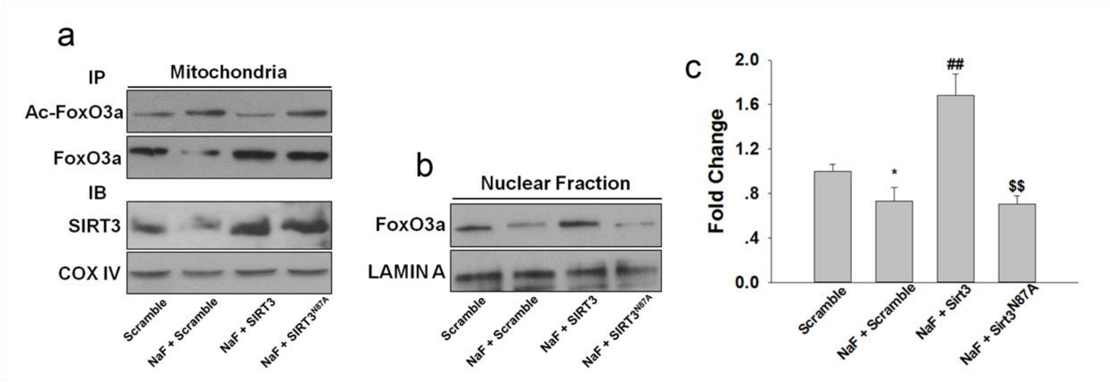

Supplementary Figure S4 Overexpression of SIRT3 enhanced SOD2 expression through the interaction with FoxO3a in NaF-treated TCMK-1 cells. TCMK-1 cells were transfected with SIRT3 expression constructs (WT or N87A) followed by exposure to NaF (1 mM) for 12 h. (a) Mitochondrial fractions were immunoprecipitated with polyclonal antibodies against FoxO3a. Interaction of endogenous SIRT3 and FoxO3a was detected by immunoblotting analysis. (b) Nuclear location of FoxO3a was measured. (c) ChIP analysis was used to examine the binding of FoxO3a to the SOD2 promoter. $^{*}p < 0.05$ versus scramble group, $^{\#\#}p < 0.01$ versus the NaF + scramble group, $^{\$\$}p < 0.01$ versus the NaF + SIRT3 group.

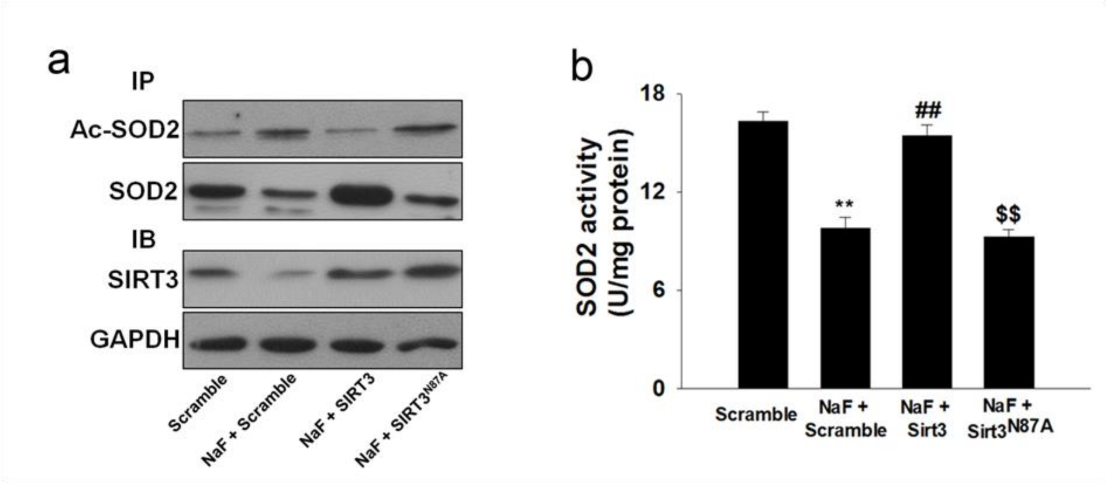

Supplementary Figure S5 SIRT3 deacetylates and activates SOD2 in TCMK-1 cells. TCMK-1 cells were transfected with SIRT3 expression constructs (WT or N87A) followed by exposure to NaF (1 mM) for 12 h. (a) Co-IP confirmed that SIRT3 de-acetylated SOD2 by directly binding to SOD2. (b) SOD2 activity was measured. $^{**}p < 0.01$ versus scramble group, $^{\#\#}p < 0.01$ versus the NaF + scramble group, $^{\$\$}p < 0.01$ versus the NaF + SIRT3 group.

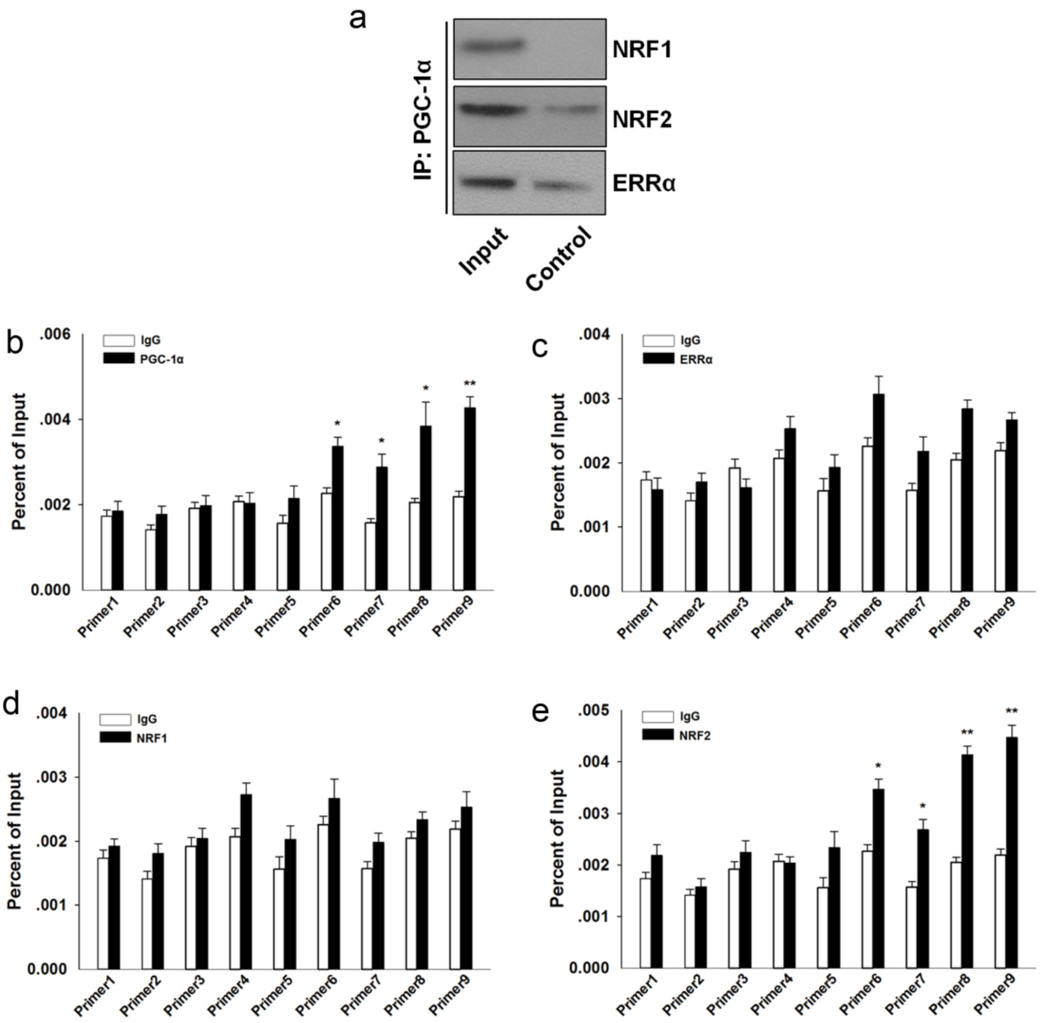

Supplementary Figure S6 PGC-1α interacts with ERRα and NRF2, and ERRα and NRF1 had little binding in the SIRT3 promoter. Cells were incubated with 3mM of NaF for 12h. (a) Cell lysates extracted from TCMK-1 cells and were immunoprecipitated with polyclonal antibody against PGC-1α. Interaction of endogenous PGC-1α with ERRα, NRF1 and NRF2 were detected by immunoblotting analysis. ChIP-qPCR was performed with PGC-1α (b), ERRα (c), NRF1 (d) and NRF2 (e) antibodies in TCMK-1 cells. Data are presented as means ± s.d. of at least three independent experiments. $^{*}p < 0.05$, $^{**}p < 0.01$.

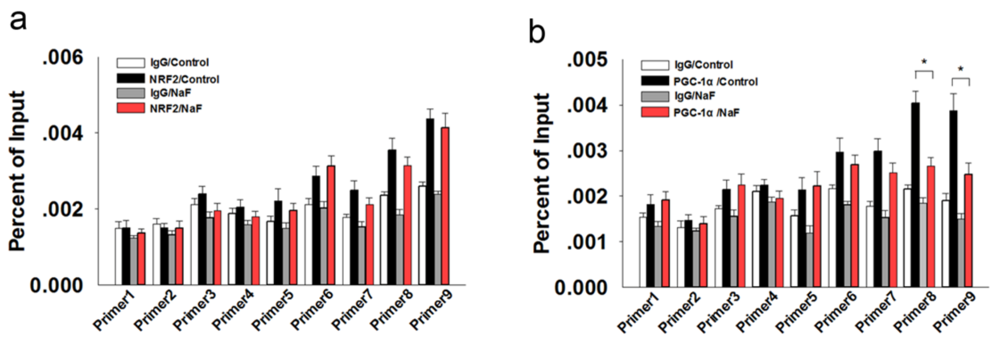

Supplementary Figure S7 NRF2/PGC-1α-Sirt3-SOD2 pathway involved in NaF-induced oxidative damage. TCMK-1 cells were incubated with 1 mM of NaF for 12h. ChIP-qPCR was performed with NRF2 (a) and PGC-1α (b) antibodies in TCMK-1 cells. Data are presented as means ± s.d. of at least three independent experiments. $^{*}p < 0.05$.

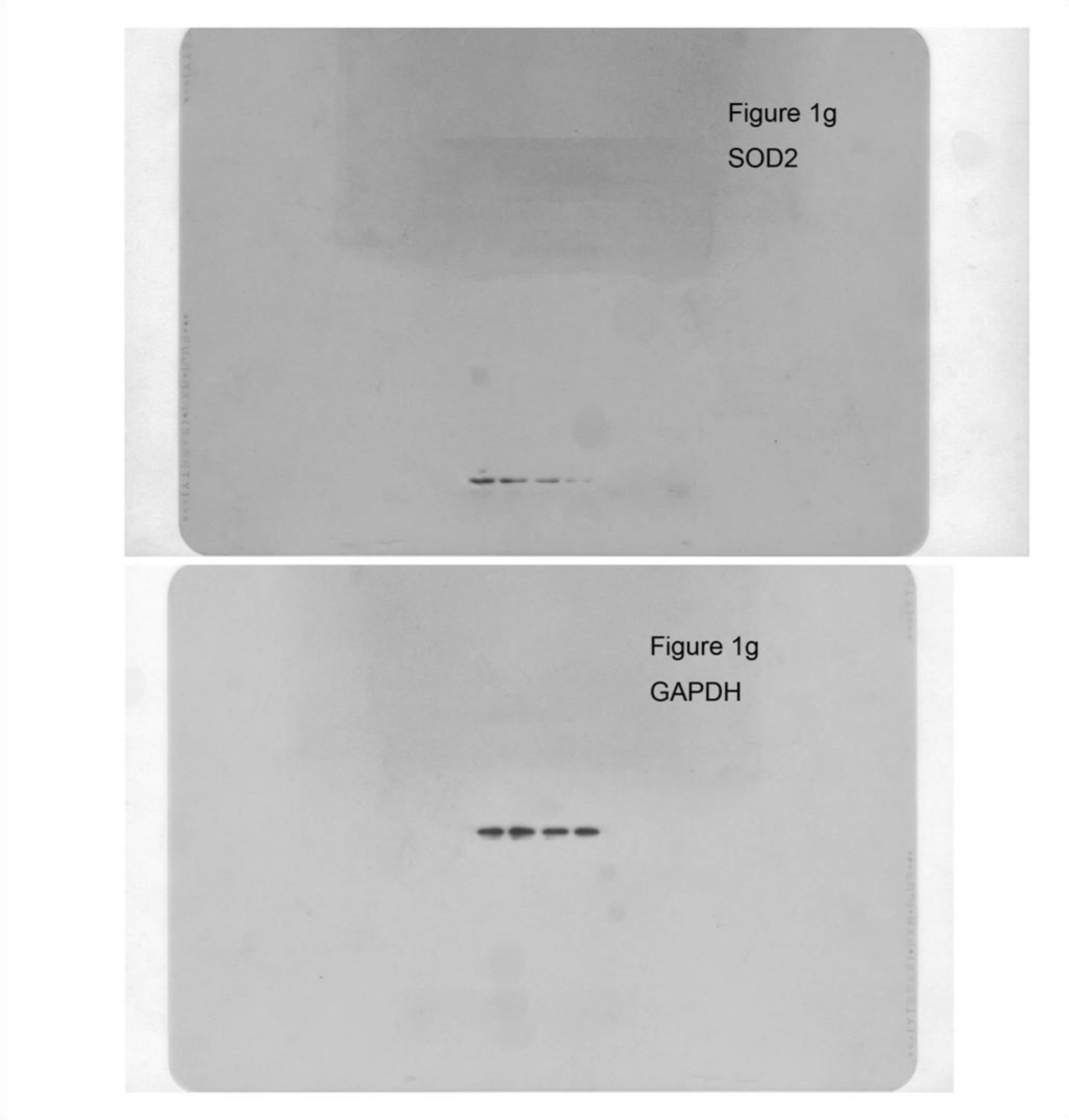

Supplementary Figure S8 The uncropped images of Figure 1g.

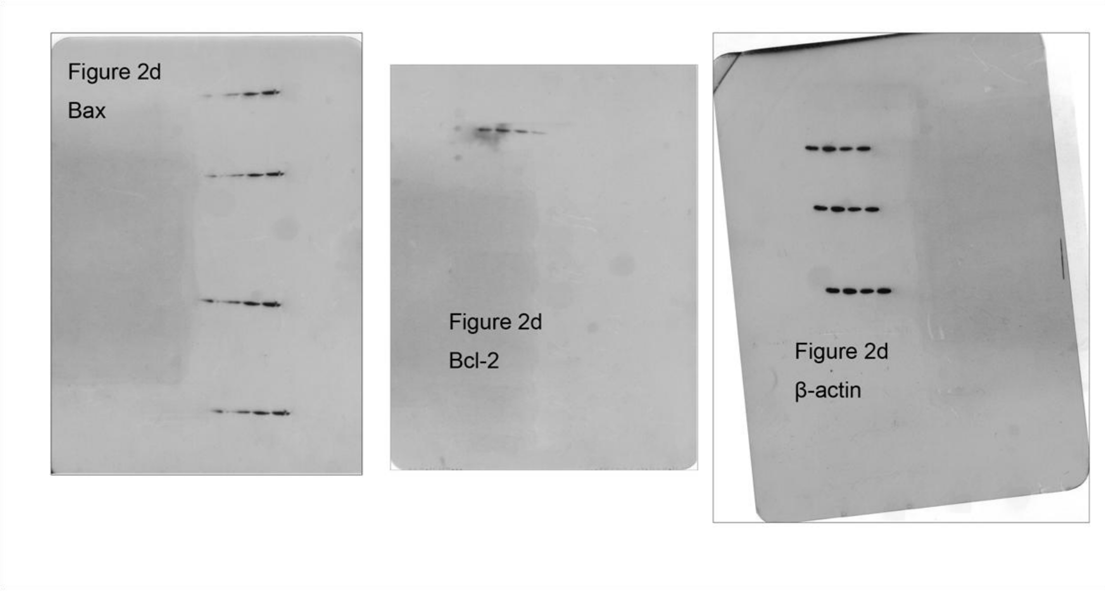

Supplementary Figure S9 The uncropped images of Figure 2d.

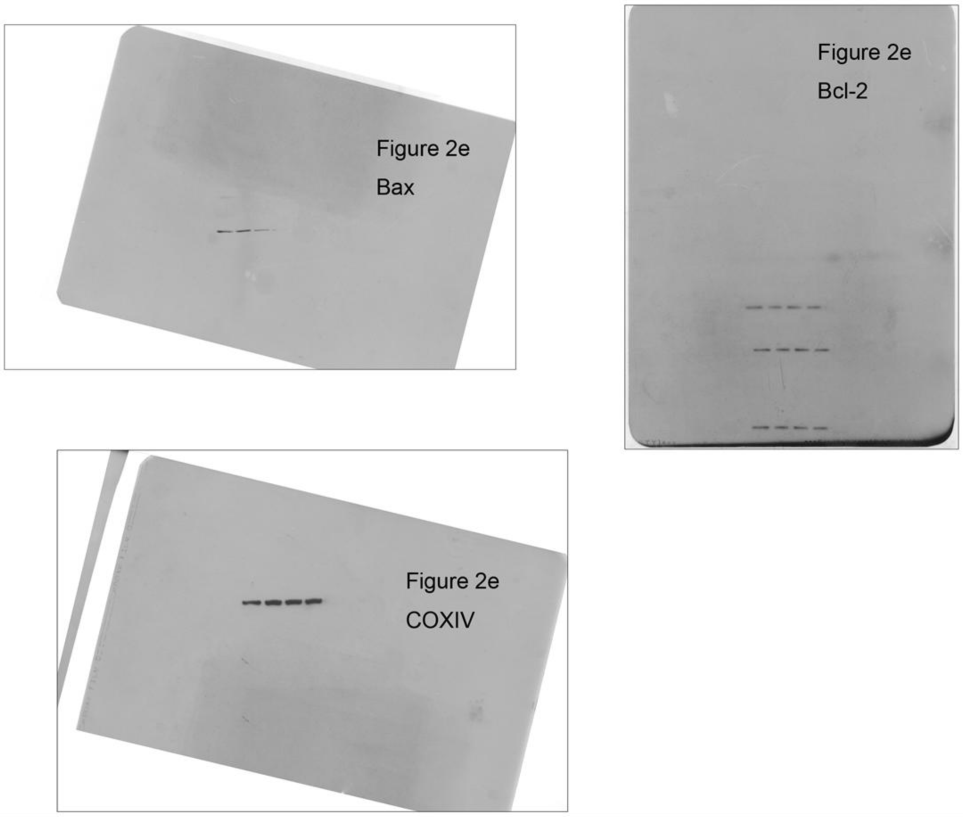

Supplementary Figure S10 The uncropped images of Figure 2e.

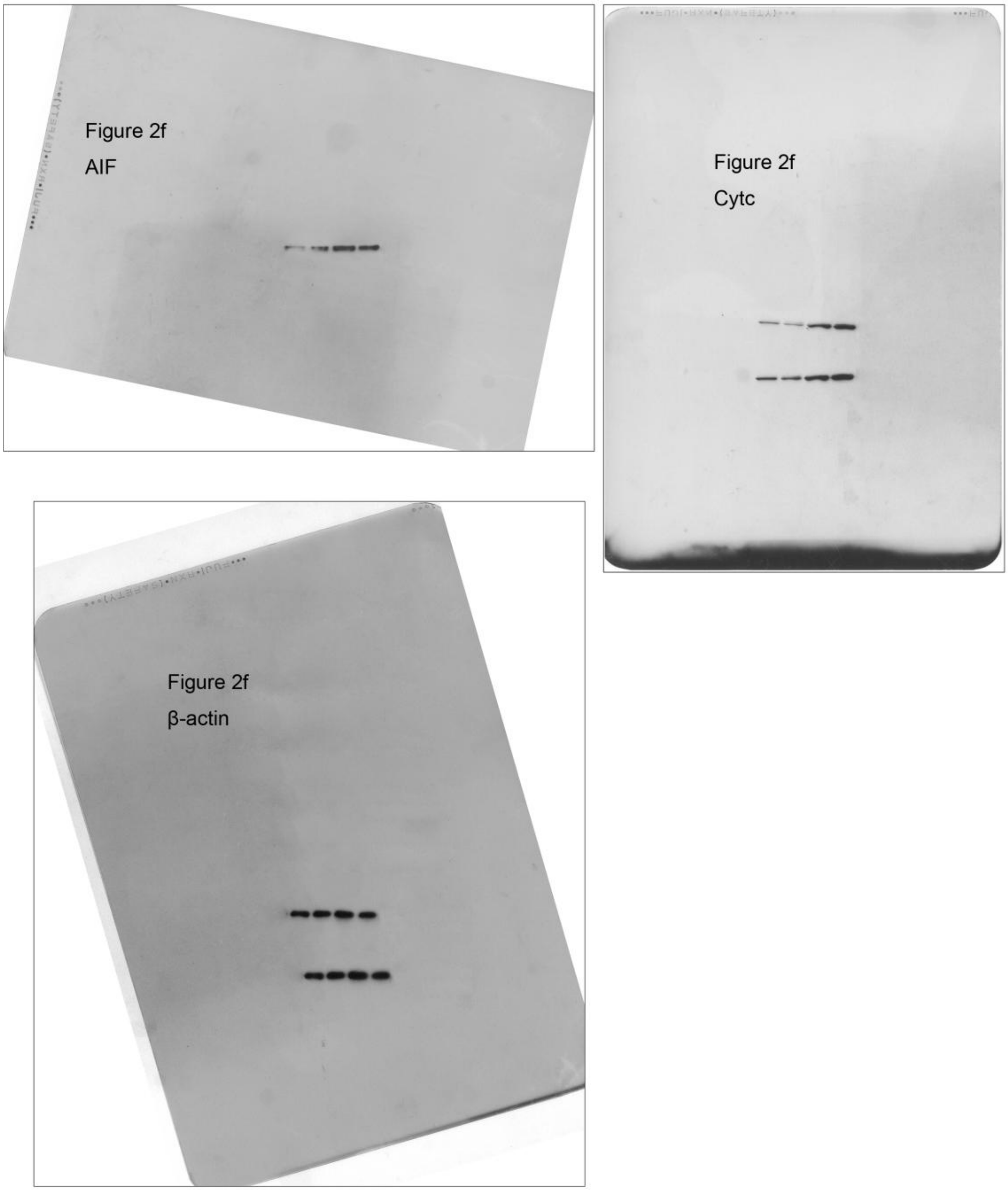

Supplementary Figure S11 The uncropped images of Figure 2f.

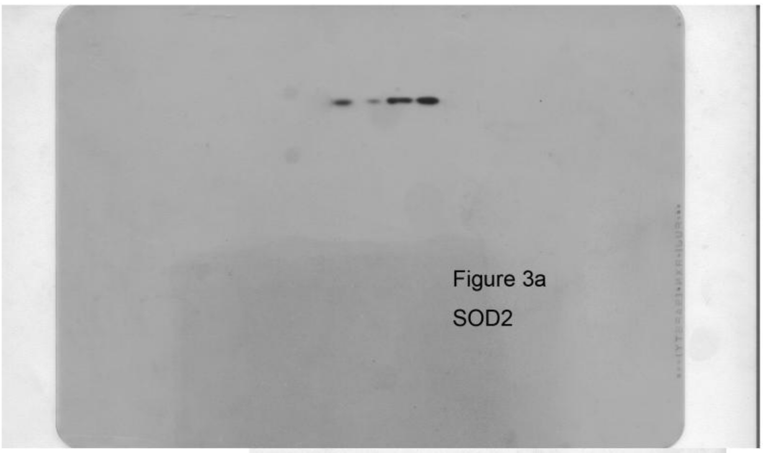

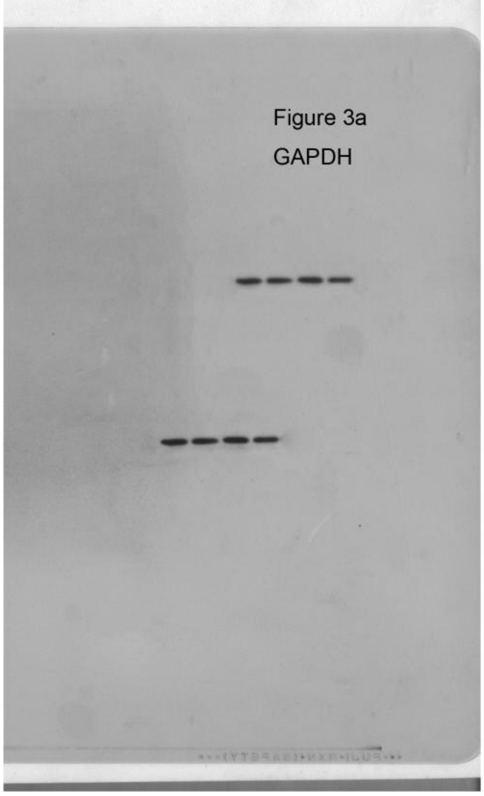

Supplementary Figure S12 The uncropped images of Figure 3a.

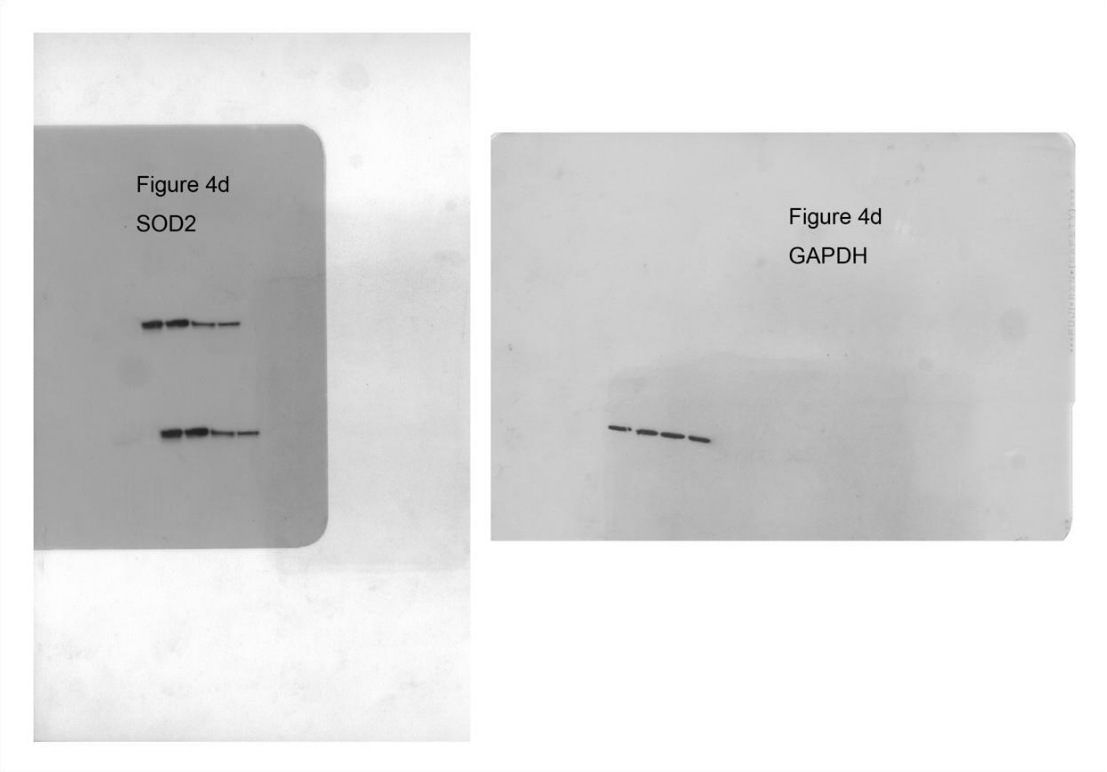

Supplementary Figure S13 The uncropped images of Figure 4d.

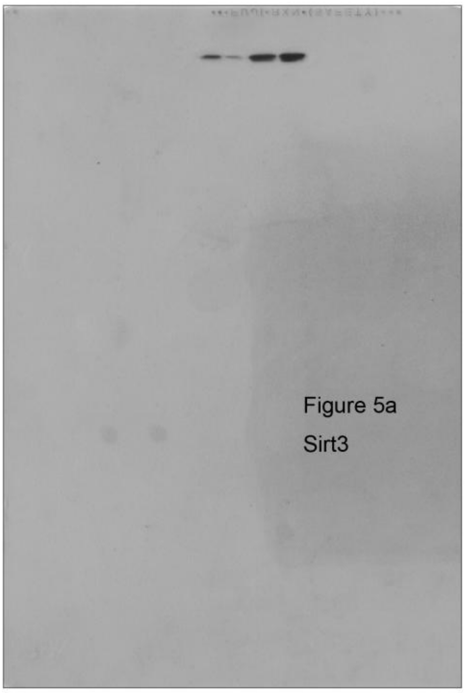

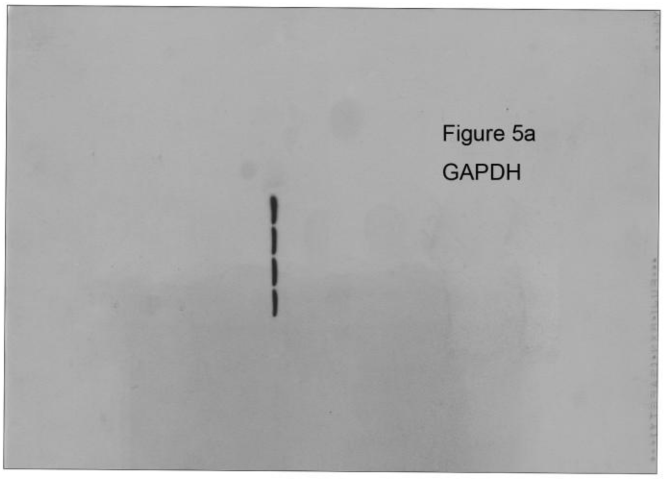

Supplementary Figure S14 The uncropped images of Figure 5a.

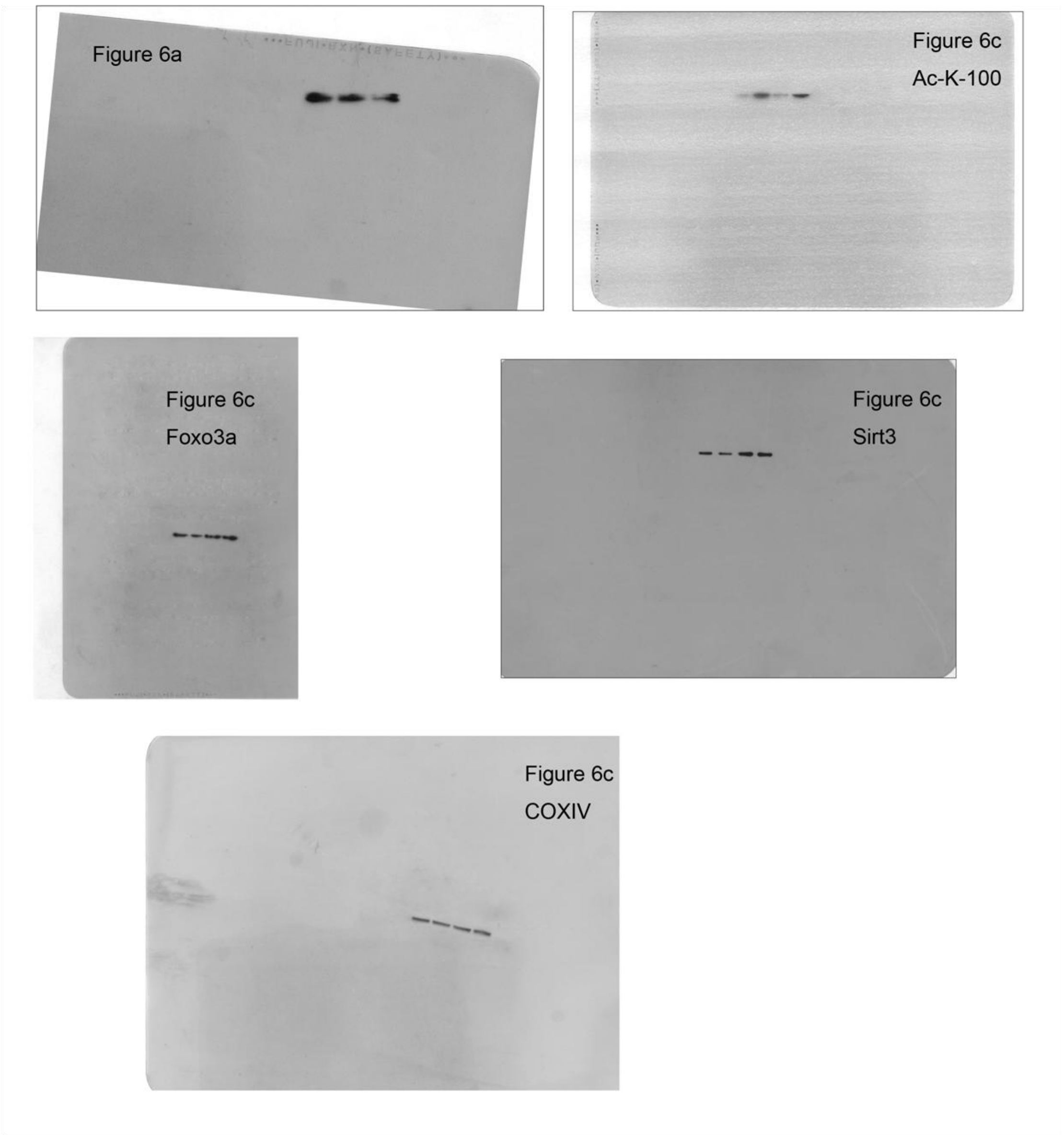

Supplementary Figure S15 The uncropped images of Figure 6c.

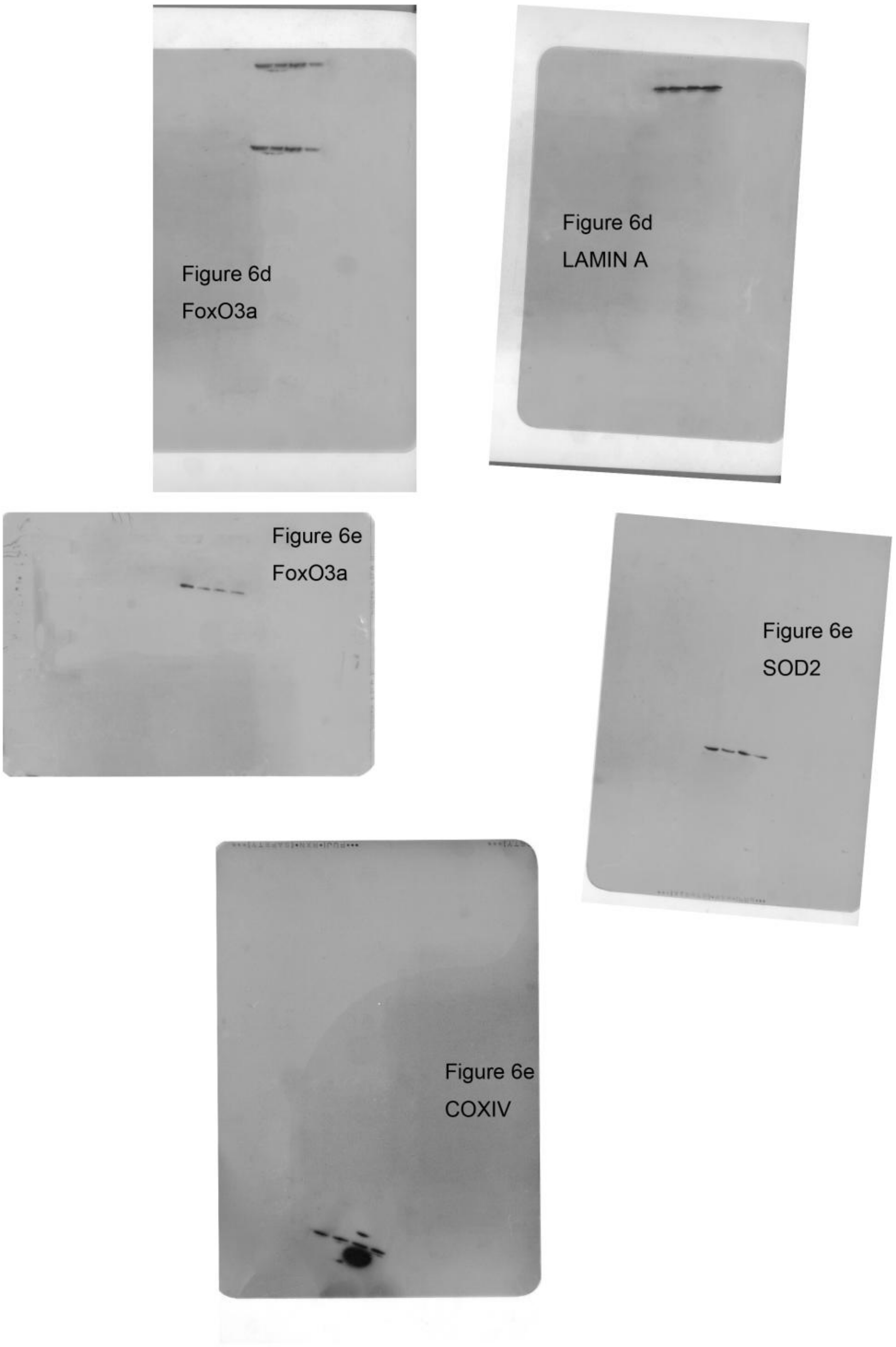

Supplementary Figure S16 The uncropped images of Figures 6d and e.

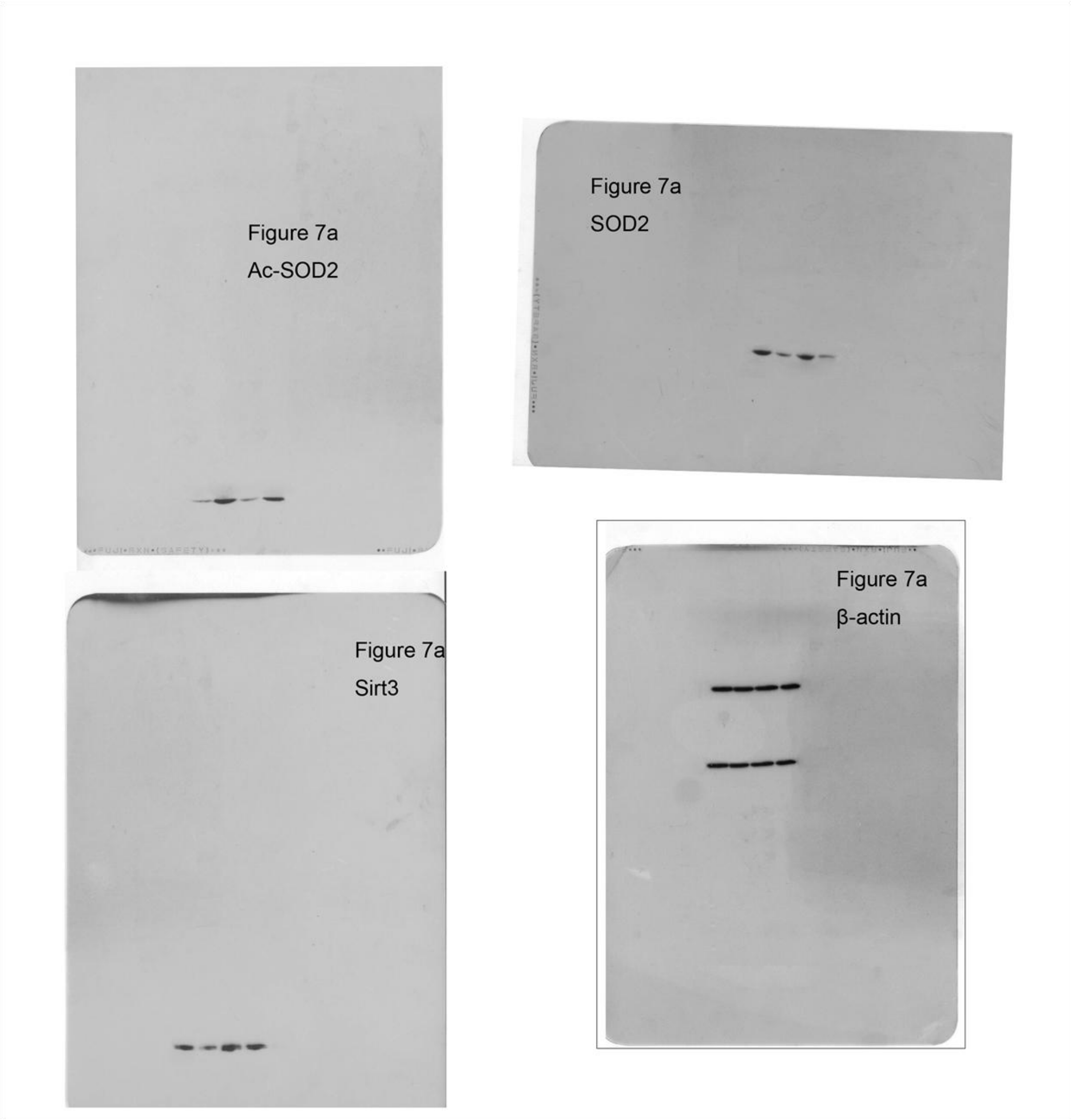

Supplementary Figure S17 The uncropped images of Figure 7a.

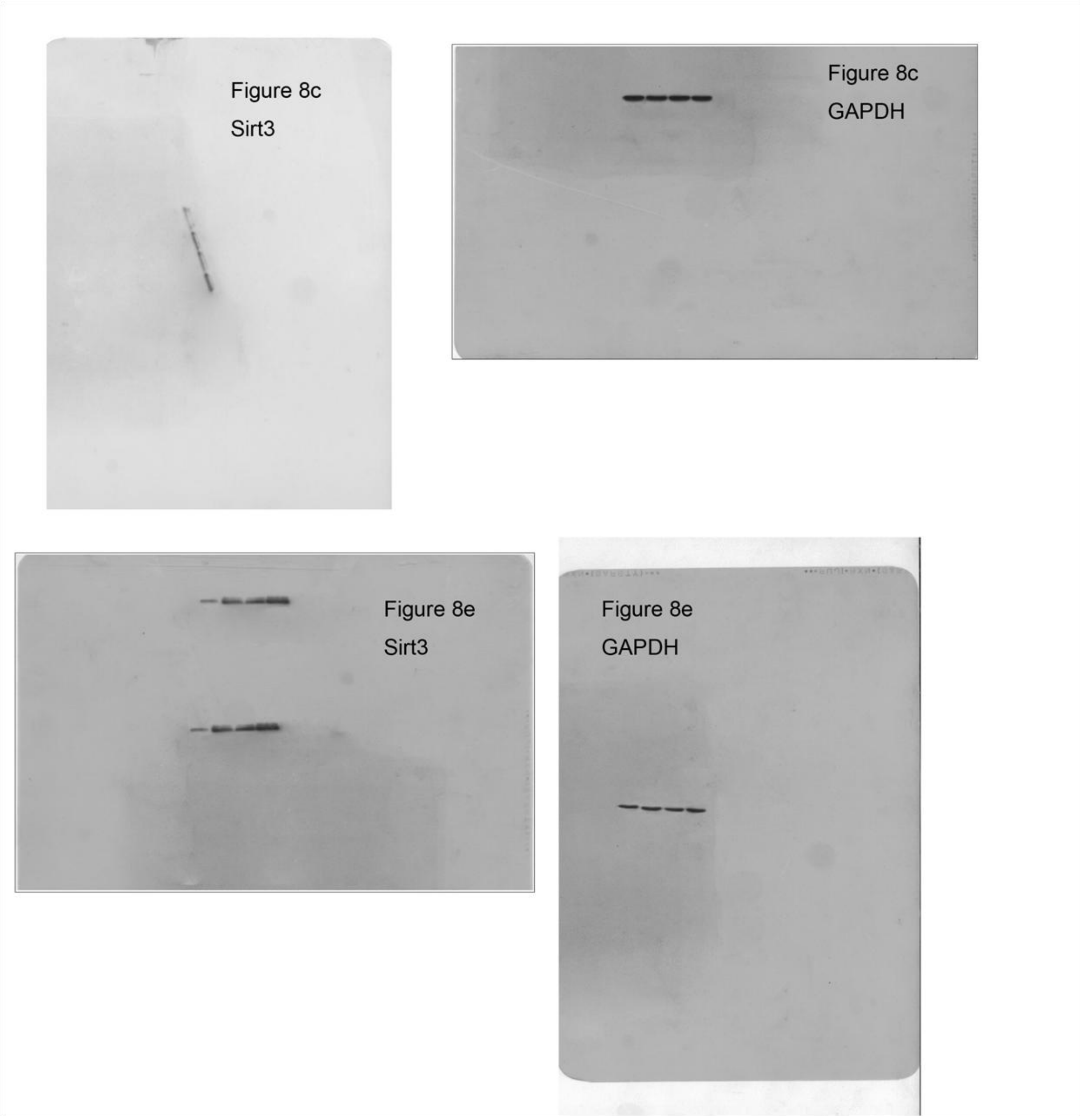

Supplementary Figure S18 The uncropped images of Figures 8c and e.

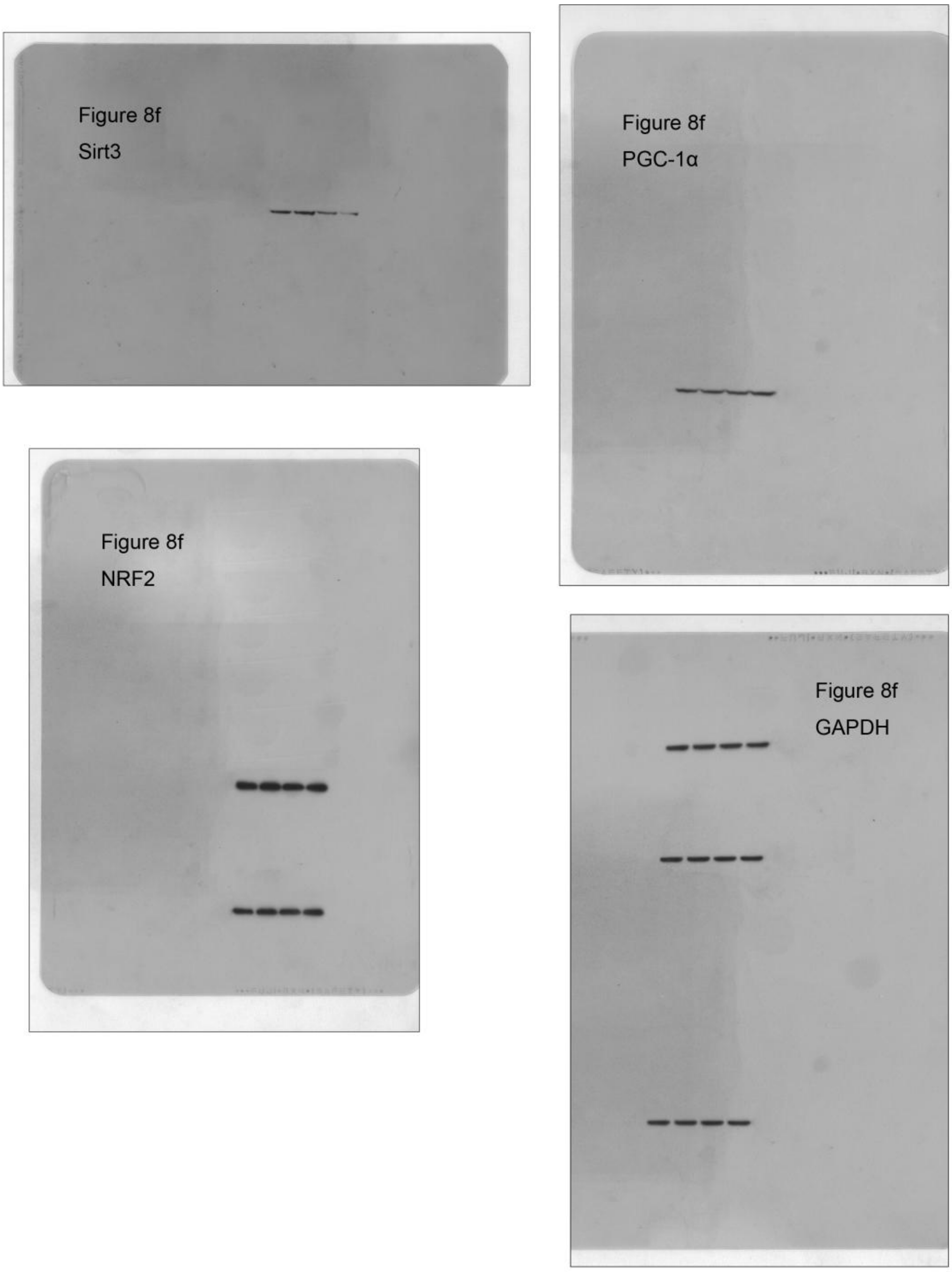

Supplementary Figure S19 The uncropped images of Figure 8f.

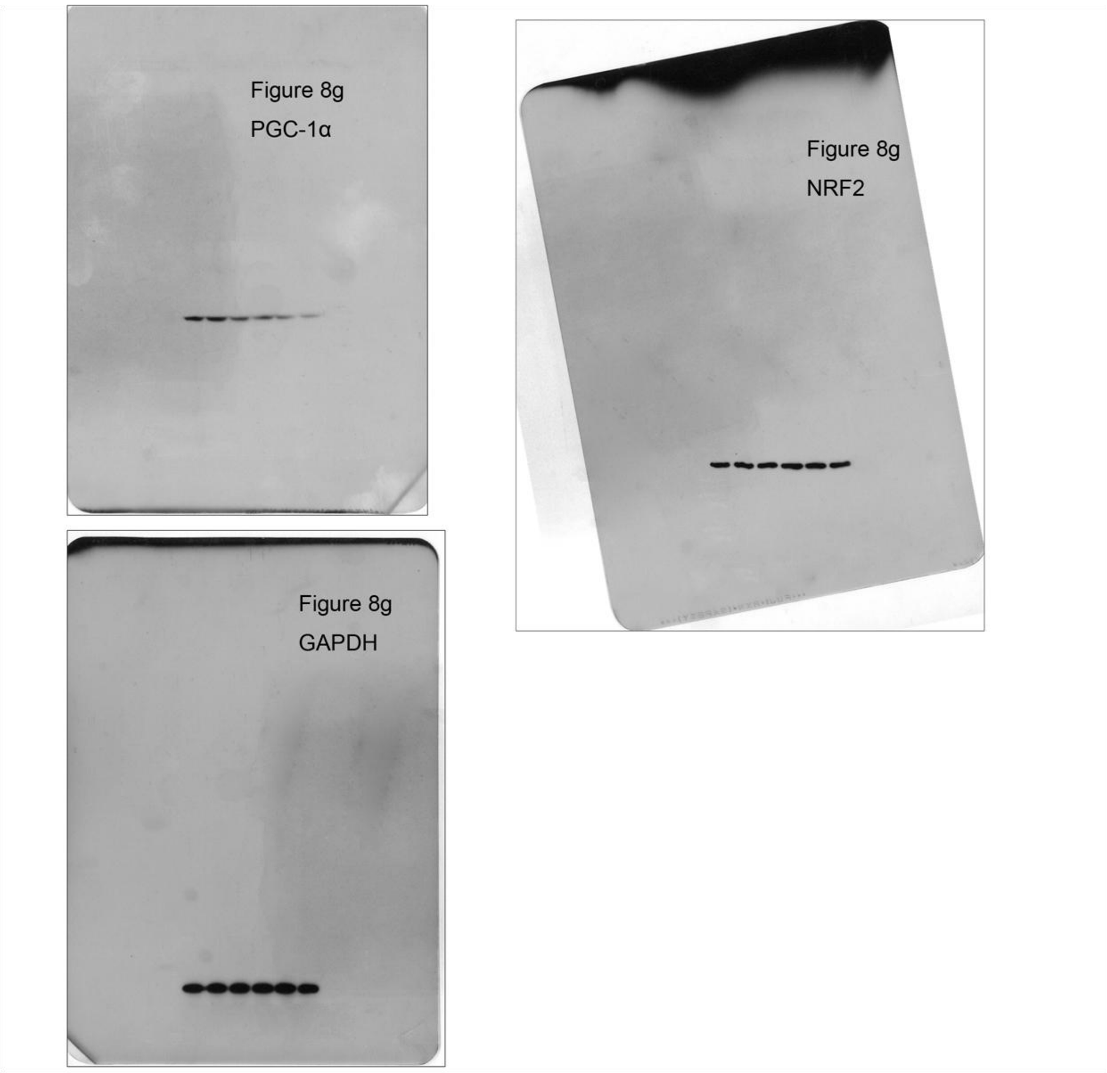

Supplementary Figure S20 The uncropped images of Figure 8g.

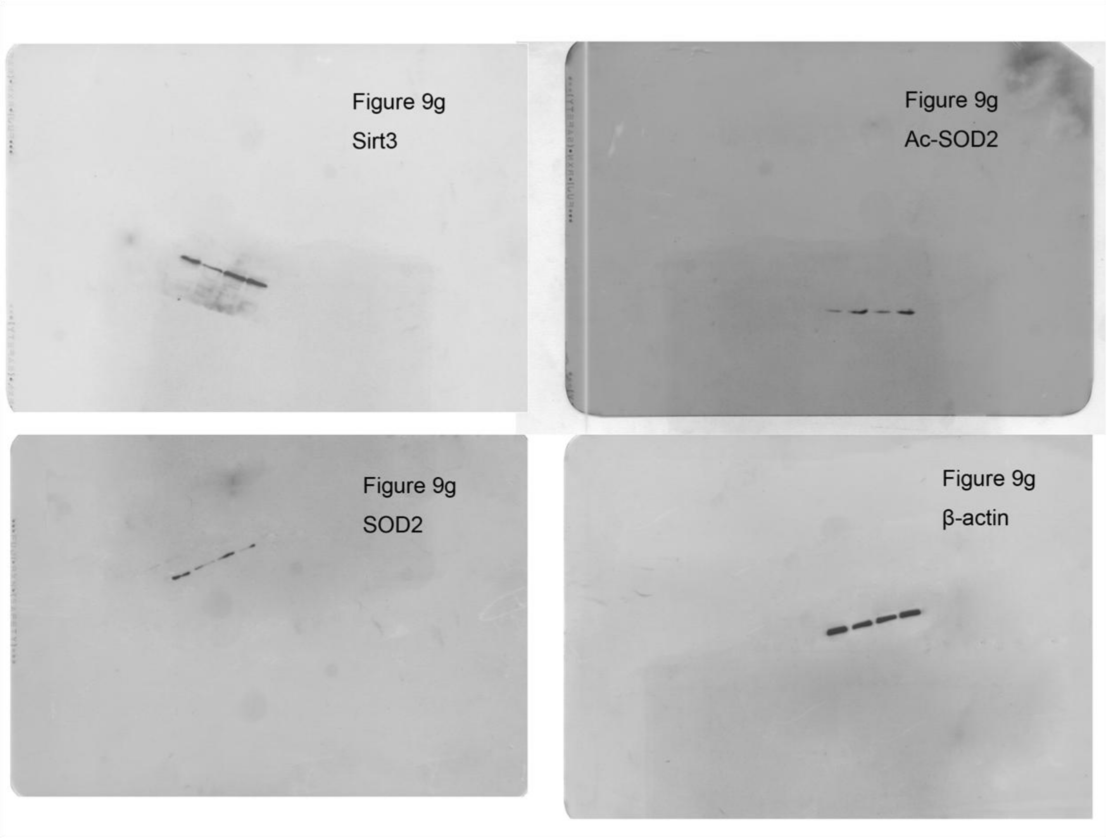

Supplementary Figure S21 The uncropped images of Figure 9g.

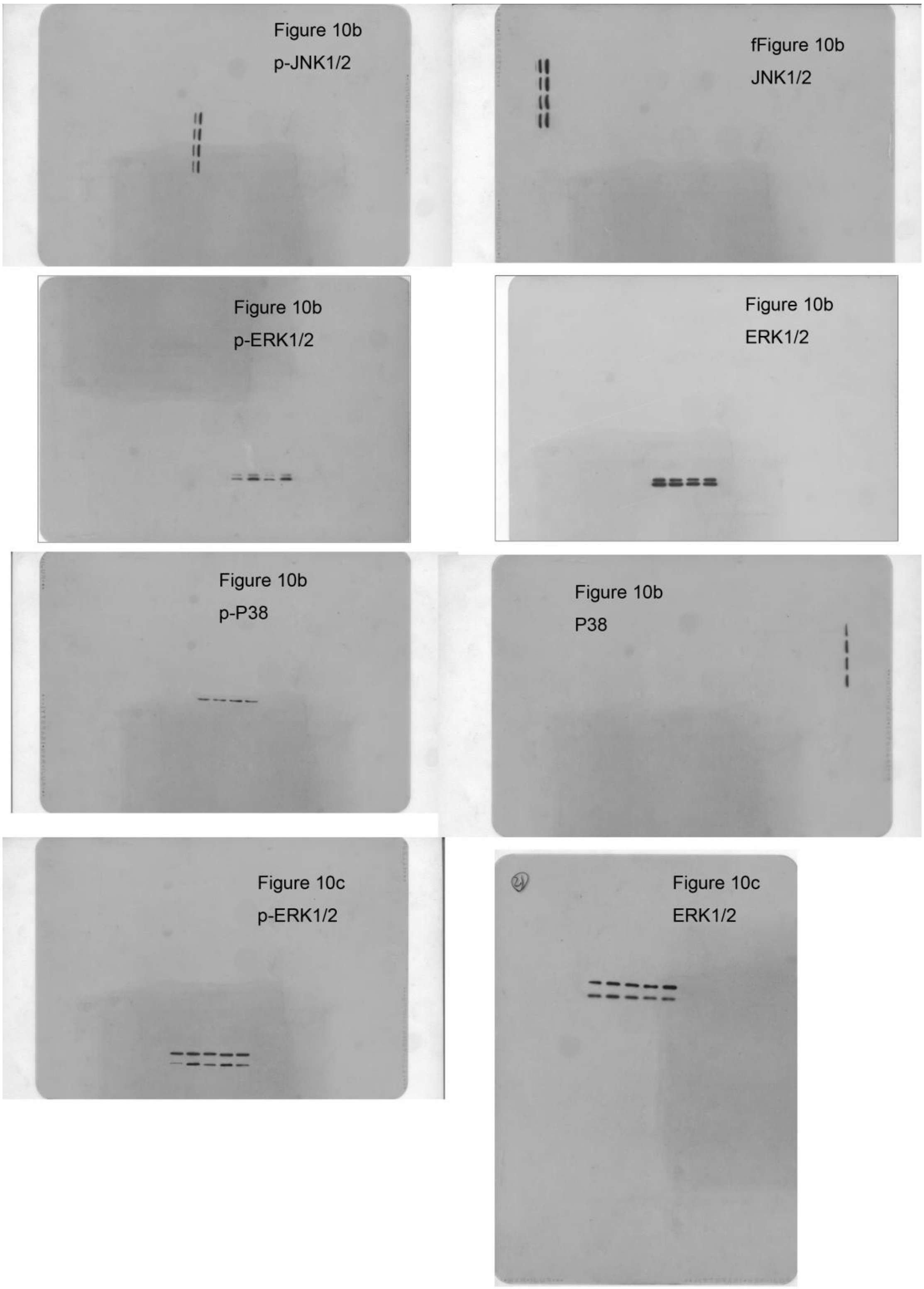

Supplementary Figure S22 The uncropped images of Figures 10b and c.
